# An alternative strategy for targeted gene replacement in plants using a dual-sgRNA/Cas9 design

**DOI:** 10.1038/srep23890

**Published:** 2016-04-01

**Authors:** Yongping Zhao, Congsheng Zhang, Wenwen Liu, Wei Gao, Changlin Liu, Gaoyuan Song, Wen-Xue Li, Long Mao, Beijiu Chen, Yunbi Xu, Xinhai Li, Chuanxiao Xie

**Affiliations:** 1 Institute of Crop Science, Chinese Academy of Agricultural Sciences, National Key Facility for Crop Gene Resources and Genetic Improvement, Beijing, 100081 China; 2 Anhui Agricultural University, Hefei, Anhui Province, 230036 China

## Abstract

Precision DNA/gene replacement is a promising genome-editing tool that is highly desirable for molecular engineering and breeding by design. Although the CRISPR/Cas9 system works well as a tool for gene knockout in plants, gene replacement has rarely been reported. Towards this end, we first designed a combinatory dual-sgRNA/Cas9 vector (construct #1) that successfully deleted miRNA gene regions (*MIR169a* and *MIR827a*). The deletions were confirmed by PCR and subsequent sequencing, yielding deletion efficiencies of 20% and 24% on *MIR169a* and *MIR827a* loci, respectively. We designed a second structure (construct #2) that contains sites homologous to *Arabidopsis TERMINAL FLOWER 1* (*TFL1*) for homology-directed repair (HDR) with regions corresponding to the two sgRNAs on the modified construct #1. The two constructs were co-transformed into *Arabidopsis* plants to provide both targeted deletion and donor repair for targeted gene replacement by HDR. Four of 500 stably transformed T0 transgenic plants (0.8%) contained replaced fragments. The presence of the expected recombination sites was further confirmed by sequencing. Therefore, we successfully established a gene deletion/replacement system in stably transformed plants that can potentially be utilized to introduce genes of interest for targeted crop improvement.

Straightforward methodologies for precisely targeted genome editing could have significant applications in both the functional characterization of plant genes and genetic improvement. In recent years, meganuclease^1^, zinc-finger nucleases (ZFNs)^2^, and transcription activator-like effector nucleases (TALENs)^3^ have been developed as sequence-specific nucleases for the introduction of targeted double-strand breaks (DSBs) in DNA to allow gene editing *via* endogenous DNA lesion repair pathways. More recently, a new RNA-guided gene-editing tool has been developed based on the clustered regularly interspaced short palindromic repeats (CRISPR)-associated nuclease system. The CRISPR/Cas9 nuclease system can be programed to target specific genomic sites with a single chimeric RNA (a so-called single guide RNA (sgRNA)), with a higher degree of flexibility for target selection than protein-guided targeting tools^4^. The CRISPR/Cas9 system has been demonstrated to facilitate genome editing in diverse species, including mammals (including humans), microbes, and plants^5^,^6^,^7^,^8^,^9^,^10^,^11^,^12^,^13^,^14^,^15^.

The gene knockouts achieved using CRISPR/Cas9 represent the earliest applications of this system because the DSBs induced by Cas9 are repaired by a non-homologous end-joining (NHEJ) mechanism, which is a type of error-prone repair pathway that can introduce short deletions or insertions during DNA repair^16^. HDR (homology-directed repair), which is an alternative means of repairing DSBs in chromosomes^17^, is considerably more attractive for engineering plant genomes^18^ because more subtle DNA sequence modifications can be achieved, including DNA correction, targeted knock-in^15^ or replacement, or any type of desired mutation. HDR-dependent targeted gene replacement or knock-in provides an unprecedented opportunity in genome editing but has been more challenging because target DSB(s) and a repair template must co-exist. Targeted genomic deletion mutations can be generated by using pairs of TALENs or sgRNAs with the Cas9 nuclease^15^,^19^,^20^. The induced double DSB lesions or deletion mutations are also a prerequisite step for targeted gene replacement. In an *in vitro* system, CRISPR/Cas9 was reported to successfully achieve HDR-mediated gene replacement in tobacco (*N. benthamiana*) protoplasts^6^. The insertion of a trait gene into a targeted DSB lesion generated by CRISPR/Cas9 via HDR has also been reported in maize, in which the DNA donor repair template was co-bombarded with Cas9 and sgRNA expression cassettes to ensure that sufficient donor copies were present in the target cells^21^. The advantage of particle bombardment strategies is that many copies of the donor template can be provided. However, the transformation copies should be handled in successive steps in the target organism, and a possible partial DNA sequence from the vector could be a contaminant for the recipient genome^22^. Stable transformation of CRISPR/Cas9^14^ and a vector containing the necessary element for genome editing via *Agrobacterium*-mediated transformation can be easily screened by identifying “transgene-free” organisms. With respect to HDR-mediated gene targeting, Fauser *et al*.^23^ demonstrated that both the nuclease and the nickase systems are efficient tools. The subtle design of the adjacent paired sgRNA/Cas9 nickase, which could have the advantage of enhanced specificity, could be used for target gene insertion, gene stacking, and knock-in when the paired sites are close each other^15^. To our knowledge, the creation of an *in vivo* targeted gene replacement event in plants with a lower risk of transgene contamination has not been reported to date.

In this work, we report the achievement of the subtle and precise deletion of MIR genes via the simultaneous delivery of a pair of sgRNAs designed to target both of the flanking regions of two MIRs: *AtMIR169a* and *AtMIR827a*. Then, we achieved the targeted replacement of a gene of interest via the stable transformation of both CRSPR/Cas9 and a DNA repair donor template, in which the same sgRNA target sites were designed to delete the plant target gene and to delete the DNA donor that facilitated HDR (Fig. 1). Our study demonstrates an alternative strategy that uses the CRISPR/Cas9 system for genome editing via gene replacement.

## Results

### Design of the dual-sgRNA CRISPR/Cas9 system

**Figure 1:**
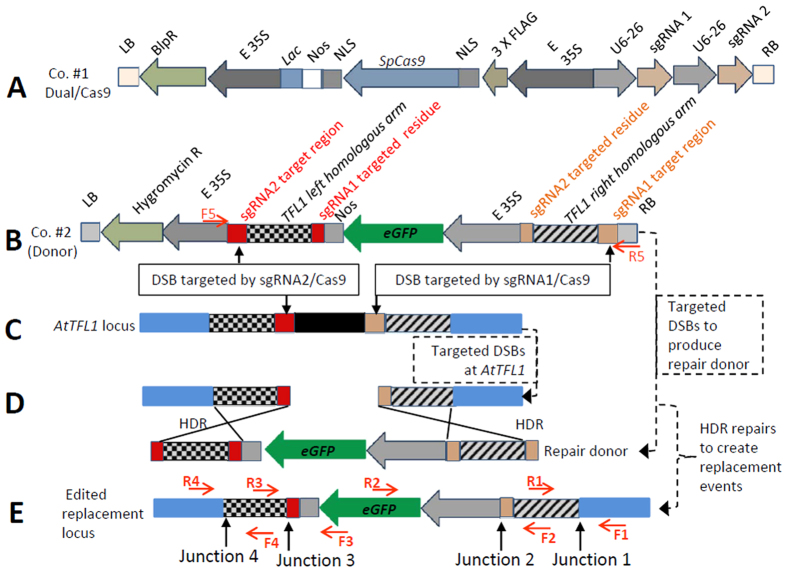
Illustration of the design of an alternative strategy for targeted gene replacement at the *AtTFL1* locus using a dual-sgRNA/Cas9 design. (**A**) The components of the dual-sgRNAs CRISPR/Cas9 constructs (construct #1). 3 × FLAG, 3 × FLAG-tag; BlpR, bialaphos resistance marker; E 35S, enhanced 35S CaMV promoter; *Lac, E. coli Lac* operator elements; LB, T DNA left border; NLS, nuclear location signal sequence (SV40 and nucleoplasmin NLS sequences were used at both ends of the Cas9 nuclease); Nos, Nos terminator; RB, T DNA right border; sgRNA, single guiding RNA; *SpCas9, Streptococcus pyogenes Cas9*. (**B**) The construct used to provide the replacement DNA donor (construct #2). *AtTFL1* left homologous arm (733 bp); *AtTFL1* right homologous arm (825 bp); eGFP, eGFP gene with an SV40 NLS sequence; F5 and R5, primer pair used to verify that the DNA repair donor had been deleted; sgRNA1 or sgRNA2 target region, the sequence designed to be targeted by the sgRNA1/Cas9 or sgRNA2/Cas9 nuclease, respectively; sgRNA1 or sgRNA2 targeted residue, the expected residue sequenced after the excision of *AtTFL1* targeted by the sgRNA1/Cas9 nuclease (as the part of the end of the homologous arm). (**C**) *AtTFL1* locus before replacement mutation. The sequence with the black and white square mosaic is the region homologous to the donor sequence; red rectangle region, the target site region of sgRNA2; brown rectangle region, the target site region of sgRNA1. The blue rectangle indicates the plant genome outside of the target region. (Note: elements indicated with the same color and shape indicated the same sequences shown in Fig. 1B–E. (**D**) Schematic illustration of the homology-directed repair activities through which the eGFP expression cassette is inserted into the targeted locus. The targeted DSBs were induced in both *AtTFL1* loci of the chromosome (**C**) and transformed with repair donor template(s). In addition, homology-directed repair gives rise to the eGFP sequence, replacing the local deleted *AtTFL1* region. (**E**) The edited replacement locus and the detecting primer pair locations. (Primers F1 and R1, F2 and R2, F3 and R3, and F4 and R4 were used as primer pairs for detecting junction sites across the designed elements.)

In the present study, to delete the targeted loci and replace the target gene region with a gene of interest, we designed a construct (construct #1) to express dual sgRNAs and the CRISPR/Cas9 nuclease to achieve targeted gene deletion activity (Fig. 1A). For target gene replacement, an additional DNA donor construct (construct #2, Fig. 1B) harbored the intended replacement gene, the homologous arms of the target locus and both recognition sites of construct #1 outside the homologous arms (Fig. 1C). The design of this study includes a target region of 255 bp from the *AtTFL1* locus (Supplementary Figure 1), which is replaced by an eGFP expression construct with an SV40 nuclear location signal (SV40 NLS) driven by an enhanced CaMV 35S promoter (Fig. 1B). The expressed dual-sgRNA CRISPR/Cas9 nuclease system leads to DSBs in the target sites of both the plant genome (Fig. 1C) and the genome-integrated DNA repair donor (Fig. 1B). DSB induction leads to HDR activation, and the repair donor DNA can then be integrated into the target site, resulting in targeted gene replacement (Fig. 1D,E; Supplementary File 1).

### Efficient creation of a heritable null mutation via targeted deletion at the *AtMIR169a* locus

**Figure 2:**
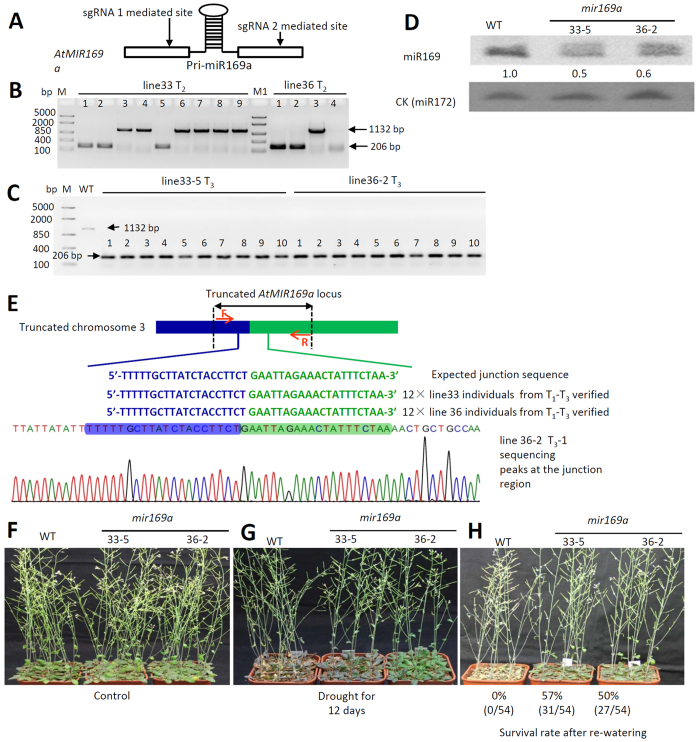
Precisely targeted and heritable null mutants with a deleted *AtMIR169a* locus (*mir169a*) were generated using the dual-sgRNA/Cas9 nuclease. (**A**) A scheme illustrating the designed targeted chromosome deletion region within the *AtMIR169a* locus. The stem-loop indicated that the region could be transcribed into pri-miR169a. The designs and the expressed dual-sgRNA targeting at two sites of *AtMIR169a* can be found in Supplementary Figure 1. (**B,C**) Screening of homologous T2 lines (**B**) and verification of the heritability and stability in T3 lines (**C**) of the targeted deletion mutation of the *AtMIR169a* locus. bp, base pairs; M1: FastRuler Middle Range DNA ladder (Thermo Scientific^TM^, Beijing); WT, wild-type. Note: The screening of T0 lines positive for the targeted deletion mutation of the *AtMIR169a* locus can be seen in Supplementary Figure 3. (**D**) Northern blotting analysis of *mir169a*, the null mutation associated with *MIR169a* deletion, using an antisense probe for the miR169 family. (**E**) Direct sequencing of the left and right arms of the targeted deletion region across the T1 to T3 generations. The junction between the left and right arms was amplified using a forward primer (indicated by the red arrow and the letter “F”) and a reverse primer (indicated by the red arrow and the letter “R”). The PCR amplicons were then cloned into a TA vector to be sequenced in reactions using the M13 R primer, which would reveal the junction sequence as the reverse complementary sequence. A total of 12 individuals across 3 generations from lines 33 (line 33 T_1_ 1×, T_2_: line 33-5 1×, and T_3_: line 33-5 10×) and 36 (line 36 T_1_ 1×, T_2_: line 36-2 1×, and T_3_: line 36-5 10×) were sequenced. The sequencing data verified the precise junction of the chromosome deletion mediated by the two sgRNAs (the sequences underlined in bold in A,B in Supplementary Figure 2 were verified to be re-joined after precision repair). (**F–H**) Improved drought resistance of the homozygous CRISPR/Cas9-edited *MIR169a* T3 (*mir169a*) plants. F: well water control; G: drought for 12 days; H: survival rate after drought treatment and re-watering for 3 days. WT, wild-type Columbia C0; *mir169a, MIR169a* deletion mutants; lines 33-5 and 36-2 were the line accession numbers.

Highly efficient target deletion is one of the key features of this design. To test this capacity, the pri-miRNA region of *AtMIR169a* (Fig. 2A) was subjected to targeted deletion using construct #1, which targets both ends of the locus (Fig. 2A, Supplementary Figure 2 A,B). We expected the underlined sequences shown in Supplementary Figure 2 A,B to be joined together after the precise repair of both DSB lesions induced by the sgRNA-guided Cas9 nuclease. The design details of the dual-sgRNA/Cas9-mediated targeted deletion and replacement are listed in Table 1, which shows the two sgRNA-targeting sequences, the chromosome regions, and the protospacer-adjacent motif (PAM) sequence.

To obtain heritable targeted deletion mutations, we screened for the targeted mutation in successive generations of dual-sgRNA/Cas9-transformed events from T0 to T3. Fifty T0 plants were screened for the targeted deletion mutation (Table 2). Twenty percent of the observed (10/50) T0 plants harbored a 934-bp deletion mutation at the *AtMIR169a* locus. Forty-four T1 lines derived from the original 50 T0 lines were screened in the T1 generation (Supplementary Figure 3). All 10 T0 mutant lines that carried the mutation passed the mutation on to the T1 generation. Two lines, 33 and 36, were selected to screen for further homozygous mutant lines in the T2 (Fig. 2B) and T3 generations (Fig. 2C, Table 2). Homozygous individuals were found in the T2 generation (Fig. 2B). To verify the inheritance of the mutation, the ten individuals from the T3 lines were genotyped again, and the mutations were reconfirmed (Fig. 2C).

To further verify the deletion of the *AtMIR169a* locus, we sequenced the mutant in the region flanking the deletion (Fig. 2E). The reconnection of the left (Fig. 2E in blue) and right chromatin arms (Fig. 2E in green) after the generation of the deletion mutation mediated by the dual-sgRNA CRISPR system was verified by sequencing 12 individuals each from the homozygous lines 33 and 36 in the T1 (1 individual), T2 (1 individual), and T3 (10 individuals) generations. Sequence data from 24 individuals in the two lines across the three generations showed that these mutations rejoined precisely at the expected site (Supplementary Figure 2A,B) and were transmitted accurately across generations. The mutation was confirmed at the transcriptional level using northern blots (Fig. 2D) probed for the *miR169* family and via drought-response phenotyping of the mutant (Fig. 2F,G,H). The hybridization signal from the miR169 probe in the *mir169a* lines was notably weaker than the wild-type (WT) signal, indicating that the level of expression of mature *miR169* family members was much lower in the *mir169a* lines than in the WT (*miR169a*) (Fig. 2D). There are 14 *miR169* family members in *Arabidopsis* (miRbase: http://www.mirbase.org/), among which *miR169a* is one of the major family members^24^,^25^. Some members of the miR169 family share the same mature miRNA sequence, and family members cannot be discriminated by northern blot analysis. Therefore, the northern blot showed a weaker signal in the deletion mutant than in the WT, but the signal was not absent. After 12 days of drought treatment (Fig. 2G), the survival rates of the plants receiving the re-watering treatments (Fig. 2H) with the deleted *MIR169a* locus, *mir169a*, were 57% (31/54 for line 33-5) and 54% (27/54 for line 36-2) (Fig. 2F–H). The *mir169a* mutant lines exhibited greater drought tolerance than the WT, for which no individual (0/54) survived within the drought stress-treated populations. This observation was consistent with the results obtained for plants bearing an *AtmiR169a*/*NFYA5* module loss-of-function mutation^24^.

### Independent verification of the deletion mutation at the *AtMIR827a* locus by dual-sgRNAs/Cas9

**Figure 3:**
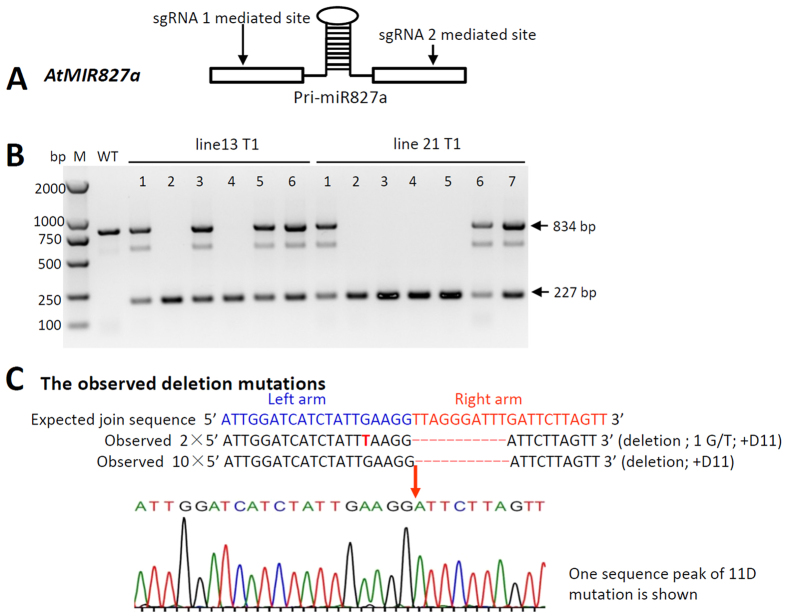
Independent verification of the deletion mutation at the *AtMIR827a* locus by the dual-sgRNAs/Cas9 nuclease. (**A**) Scheme illustrating the designed targeted chromosome deletion region at the *AtMIR827a* locus. The stem-loop indicated that the region could be transcribed into pri-miR827a. (**B**) Screening of homozygous T1 lines of the targeted deletion mutation of the *AtMIR827* locus. bp, base pairs; M: BM2000 DNA maker (Biomed^TM^, Beijing, China); WT, wild-type. The bands between 834 bp and 227 bp were unknown non-target amplification products. (**C**) The observed targeted deletion mutation of *MIR827* between the two sgRNA-mediated sites. A deletion mutation occurs as expected when the left arm (sequence in blue), which contains a DSB induced by sgRNA1, and the right arm (sequence in red), which contains a DSB induced by sgRNA2, are joined by non-homologous end-joining. G/T, a G to T substitution; +D11, a deletion that is 11 bp longer than the expected deletion mutation.

To confirm that this gene deletion could be reproducible, an independent verification was made by creating deletion mutants of *AtMIR827a*, which is another important MIR gene. A region of approximately 607 bp (Table 2) that contains the *pri-mir827a* region (Fig. 3A) was targeted for deletion with two Cas9 sgRNAs. Among 50 T0 Cas9 transgenic-positive plants, 12 deletion mutations (24.0%) were identified, 11 of which could be passed on to the T1 generation. Even in the T1 generation, homologous lines could be identified. In this study, from line 13 and line 21 (Fig. 3B), 2 individual plants of line 13 (line 13-2 and -4) and 4 individual plants of line 21 (line 21-2, -3, -4 and -5) were identified as harboring a homologous null mutation that included the *MIR827a* target region deletion. The phenotypes of four independent homozygous lines were verified (data and results will be reported in a separate paper on miR827 functional studies). Among those 12 deletion mutations, an 11-bp deletion (+D11) in the right arm near the sgRNA2-targeting site was found frequently and resulted in the deletion of 618 bp rather than the targeted 607 bp (Supplementary Figure 4). This experiment indicates that the null mutation generated via targeted deletion by the dual-sgRNA CRISPR/Cas9 system was highly reproducible and highly efficient.

### Targeted gene replacement beyond targeted deletion by the simultaneous stable transformation of a DNA repair donor template

**Figure 4:**
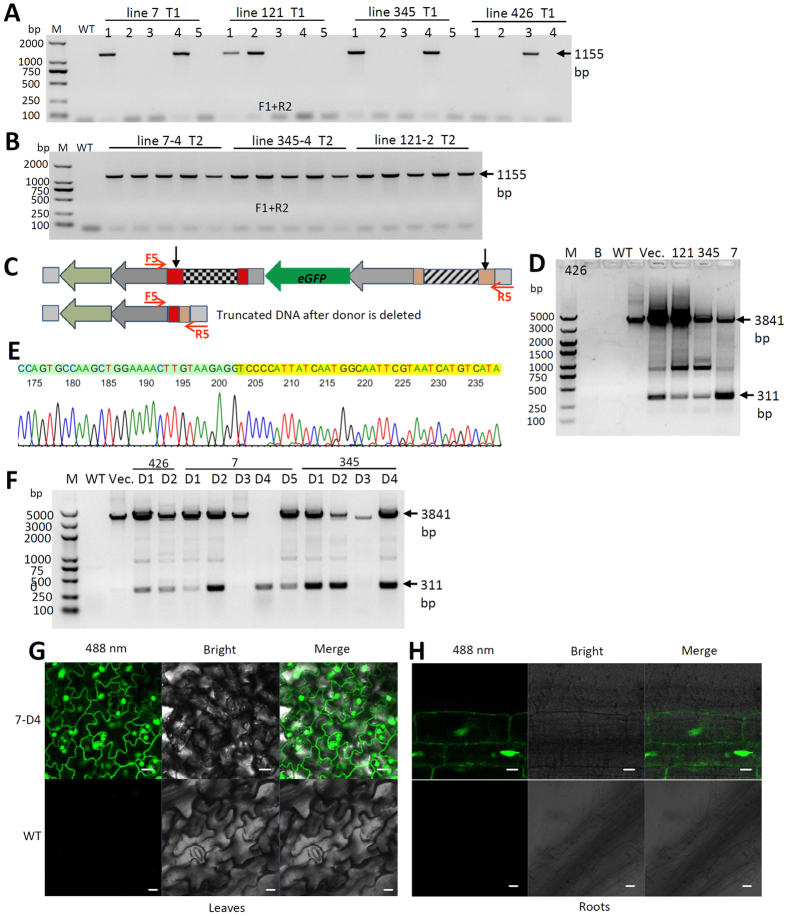
Targeted gene replacement of the *AtTFL1* region with an *eGFP* expression cassette. (**A**,**B**) PCR screening for target region replacement in the T1 (A) and T2 (B) generations using primer pair F1 and R2 (Fig. 1E). The 1,155 bp amplicons are positive replacement events. (**C–F**) Experimental evidence that the DNA donor had been deleted. (**C**) Illustration of the targeted deletion region. F5 and R5 and red arrows (Fig. 4C is same as Fig. 1B) indicate the detecting primer pair. Black vertical arrows indicate the targeted sites. (**D**) PCR verification (primer pair F5 & R5) that the DNA repair donor template was deleted. M, BM5000 + 1.5 K DNA marker; B, blank control; WT, wild-type; Vec., Vector plasmid DNA of donor template; 121, 345, 7, and 426 are four positive individuals from the T0 generation. The 3,841-bp amplicon, the amplicon harboring the DNA donor template before deletion; the 311-bp amplicon harboring the donor template had been deleted and probably supplied for gene replacement. The bands other than the 3,841-bp and 311-bp bands were unknown non-target amplification products. (**E**) Sequencing validation that the re-joining junction site of the DNA repair donor template had been cut (see details in Supplementary File 12). (**F**) Screening of individuals in which the DNA donor was deleted. 426, 7, and 345 were the lines in the T1 generation corresponding to Fig. 4A,D and D1–5 were the selected individuals from these lines that were identified as replacement-positive with the DNA donor clearly removed. M, BM5000 DNA marker. The amplicon sizes were same as Fig. 4D. (**G**,**H**) The expression of the replaced *eGFP* expression cassette in leaves (**G**) and roots (**H**) in living plants. 7D4, positive for the replacement event, but the DNA donor was absent after replacement. 488 nm, 488 nm excitation wavelength on the confocal fluorescence microscope; bright, bright-field microscopy; merge, a merged 488-nm fluorescence and bright microscope image. WT, wild-type. Note: an SV40 NLS (nuclear localized sequence) had been added before the eGFP coding region (the detailed sequence can be seen in Supplementary File 1). Thus, the expressed eGFP protein accumulated primarily in the nuclear region. Scale bar, 10 um.

To address whether we could employ targeted gene replacement beyond gene deletion, a DNA donor (construct #2) and the dual-sgRNA CRISPR/Cas9 system (construct #1) were stably co-transformed into plants using *Agrobacterium*. The T0 plants were screened by PCR by using the primer pair F1 and R2, with F1 binding to the local chromosome outside the intended site and R1 binding inside the replaced gene (Fig. 1E). In total, 500 T0 individuals with positive co-transformation events were identified. Then, four independent replacement events (lines 7, 121, 345 and 426) were found in T1 (Fig. 4A), yielding an efficiency of 0.8% for target gene replacement beyond deletion (Table 2). Three of these lines (line 7-4, line 345-4 and line 121-2) were identified as homologous targeted replacement lines (Fig. 4B). Primer pairs that bind to the target local genome outside the replacement region (primers F1 and R4 in Fig. 1E) in combination with primers that bind within the replaced region (primers R2 and F3 in Fig. 1E) could identify the replacement events. Then, the entire segment containing these events was PCR amplified using F1 and R4 to analyze the four junction sites (Fig. 4E) and verify the replacement events based on the presence of 4 exact junction sequences (Supplementary Figure 5A-E, Supplementary Files 2–5) along with the full-length sequences of the targeted replacement region. The sequencing data (Supplementary File 2–5) and the full sequences (Supplementary File 1) are provided.

To obtain additional supporting evidence, the targeted deletion that supplied the DNA donor was also analyzed (Fig. 4C–F). PCR screening of the positive gene replacement plants was used to analyze whether the DNA repair donor and its target homolog arm had been deleted to facilitate cellular HDR (Fig. 4C,D). We found that most of the individuals harbored an allele in which the DNA donor had been deleted (Fig. 4D). We also implemented sequencing to confirm the deletion and found the re-joining junction site after the DNA donor had been deleted among all 4 biological replicates (Fig. 4E & Supplementary Files 6–9). Among the T1 generation of the individuals in which the DNA donor was deleted and the replacement was positive, we looked for individuals in which the DNA donor was deleted by analyzing *eGFP* expression. Of 11 individuals from the T1 generations of lines 426, 7 and 345, (Fig. 4F), the DNA donor template harboring the eGFP expression cassette was absent in the original locus in individual 7-D4. We further examined the eGFP expression of 7-D4 in the leaves (Fig. 4G) and roots (Fig. 4H) of the plants that underwent gene replacement events in which the DNA donor of eGFP had been removed for HDR repair. Therefore, eGFP expression should only result from eGFP expression after targeted gene replacement because previous sequencing results indicated its existence in the intended region (Supplementary Figure 5A–E, Supplementary File 2-5). The expression signal of eGFP was detected primarily in the nuclei of cells in both the leaves and roots (Fig. 4G,H) due to the presence of an NLS within the eGFP expression cassette (See Supplementary File 1). Thus, the evidence from the target locus, the DNA donor region and the replaced eGFP expression cassette, along with a series of sequencing results, demonstrated that targeted gene replacement was successfully achieved using this strategy.

## Discussion

### Targeted gene replacement

Targeted gene replacement, also known as targeted gene knock-in and gene correction, enables precise DNA integration at a desired genomic location. Gene replacement was first achieved in an *in vitro* system of *N. benthamiana* protoplasts^6^. Targeted gene insertion into the targeted locus via HDR was also reported in maize through DNA bombardment^26^. Comparatively, our DNA repair template was also provided through stable transformation. The advantage of stable transformation is that the donor is from a stable system in which the DNA template in the target organism may be more complete and the residual sequence of the replacement can be exactly traced and excluded by homologous recombination during backcrossing in later generations to produce “transgene-free” individuals^14^. The shortcoming of our system was that limited donor copies could be provided in the target cells, yielding a lower frequency. However, the obtained frequency of 0.8% is still satisfactory for most plant studies. In our experiment, the sequencing results for the T1 generations provided strong experimental evidence for the identification of replacement events (Supplementary File 2–5).

Strategies for stable transformation that use both CRISPR/Cas9 and a DNA donor have also been used to generate targeted mutations in *Arabidopsis*^15^. However, the experimental systems and the key objective of our study were different. In our experimental system, the two constructs (dual-sgRNA CRISPR/Cas9 (construct #1) and DNA donor template (construct#2)) were independent constructs and were co-transformed into plants using double resistance selection systems. The dual-sgRNA CRISPR/Cas9 nuclease activity was provided by the expression of construct #1, which could create double DSBs and result in the deletion of the desired region in the plant genome. We simultaneously deleted the DNA donor (transformation of construct #2) to supply the DNA repair donor templates. Moreover, our system employed two different recognition sites, which can enable us to achieve gene replacement but not gene insertion. Schiml *et al*.^15^ embedded the CRISPR/Cas expression system and the DNA insertion donor into a single construct. In addition, the flanking homologous arms at both ends of the target sites were designed based on the same principle required by HDR^6^,^15^,^17^,^26^. Moreover, the inclusion of the paired nickase was one of the key objectives of the target insertion in the reported study^15^. The paired nickase design was used to create double nicking to improve the mutation specificity and reduce off-target effects^15^,^27^,^28^. The nickase activity resulted from an inactivating RuvC-like domain (D10A) and the effective activity of the HNH domain in the nuclease could ensure that only one DNA strand was cut^4^. The subtle design of the adjacent paired-sgRNA/Cas9 nickase could be used to achieve target gene insertion^15^ with fewer off-targets effects, but the flexibility of targeted gene replacement will be limited by the length between the double nicks.

### Length of targeted deletions or replacements using the dual-sgRNA CRISPR/Cas9 system

We successfully deleted a target gene segment of ~600–950 bp with high efficiency in plants. Our parallel studies of *Zea mays*, which is an important cereal crop, also showed that the intentional deletion length could be flexible, ranging from 1 to 300 kb (data not shown). No prior experimental evidence indicates that a larger target DNA segment can be deleted. However, a previous study in a mammalian system described intentional deletions ranging from 1.3 kilobases (kb) to greater than one megabase (1 M bp) in length^29^. Another study excised a heterozygous 30 Mbp fragment of human chromosome 15 in an otherwise haploid cell line to create a fully haploid cell line to study gene function^30^. Therefore, the CRISPR/Cas9 system should be a versatile tool for creating flexible deletion mutations ranging from 1 bp to an entire chromosome arm. The ability of a system to tolerate deletions of any length may be constrained by its biology, as deletions could have lethal effects unrelated to the use of the CRISPR/Cas9 system. Using CRISPR/Cas9 technology, we might create knockout mutants by deleting partial or entire genes. In additional to the targeted deletion, the length of gene replacement might depend on the design of the targeted knock-in^31^,^32^,^33^, in which some sequences were replaced. We anticipated that a large sequence could be manipulated as a genome-editing tool for deletion, knock-in and replacement in gene function studies and for crop genetic improvement^34^. We also believe that genome-editing tools that can handle large sequences with more versatile applications could be explored in the near future.

### Deletion of MIR genes

MicroRNAs are small endogenous RNAs that are approximately 21 nt in length and target mRNAs for cleavage or cause their translation to be repressed, thereby playing an important role in regulating gene expression^35^,^36^. Two important MIR genes that are associated with adaptation to multiple environmental stresses were identified in our previous studies^24^,^25^,^26^,^37^,^38^. Mutants have been critical for deciphering the interactions of miRNAs with their targets and the roles of miRNAs in biological processes^36^. Null mutations, or so-called gene knockouts, are valuable because they provide direct insights into the functions of gene products in an organism^39^. Large-scale T-DNA insertion mutant screens and efforts to saturate the *Arabidopsis* genome with null mutations have provided many valuable mutants for research worldwide^39^,^40^. However, it is not practical to generate a mutagenized population large enough to ensure that every gene has been mutated^39^. Furthermore, screening for mutations of interest in collections of random mutations is laborious. A further complication is that functional mature miRNAs are processed by a complex from pri-miRNA regions^35^,^36^; thus, a mutation within this short pri-miRNA region could lead to null mutations for MIR genes. There is therefore a need to further develop mutant resources for MIR genes to overcome this limitation of miRNA studies, even in model plants such as *Arabidopsis*. The resources available for the study of miRNAs in crop species are even more limited, although progress is being made in this area. In this study, *AtMIR169a* and *AtMIR827a* were selected to demonstrate the creation of null mutant lines via gene deletion for further functional characterization. The deletion of a MIR gene via the delivery of dual-sgRNA CRISPR/Cas9 is also much more practical than the single-sgRNA CRISPR/Cas9 system because it is very difficult to find a PAM sequence located close to an approximately 21-nt miRNA or miRNA* region. Even if a PAM sequence is present at the exact location, previous reports^4^,^11^ and our recent data from maize indicate that most of the mutations induced by the single-sgRNA CRISPR/Cas9 system are 1-bp deletions. The miRNA families share common mature miRNA sequences or have 1–3 nt sequence differences^35^,^36^. Thus, the single-sgRNA CRISPR/Cas9 system might mutate one miRNA but create another family member or a new miRNA. Therefore, deleting genes by using the dual-sgRNA CRISPR/Cas9 system, which results in regional deletion, should be much more valuable for MIR genes.

### Inheritance of mutations

A previous report demonstrated that the targeted mutations created with CRISPR/Cas9 were stable and passed to subsequent generations by classical Mendelian inheritance, although the majority of the mutations were somatic in early generations in *Arabidopsis*^41^. Our mutation efforts with three other genes in *Arabidopsis* also support these findings related to somatic mutation. However, the inheritance of the targeted mutations in maize differs, as most of these mutations tend to be generated in the embryonic stage and are therefore heritable. In the present study, we bypassed somatic mutations by selecting mutants that segregated in a 1:1 ratio of WT/mutant plants when screening for stable homozygous mutants that could be passed on to the next generation. During *mir169a* homozygote mutant screening, lines 33 and 36 from the T1 generation (Supplementary Figure 3) were selected for further screening and studies of this 1:1 ratio pattern. Lines 8, 32, and 42 were also verified as embryonic mutations that could be stably passed on to subsequent generations.

### Non-designed mutations and off-target effects

Our objective was to obtain deletion mutations or replacements between the dual-sgRNA targeting sites, but mutations other than the designed mutations could also be induced by either an sgRNA or dual-sgRNAs. To analyze the possible mutation patterns, we sequenced both target regions of the dual-sgRNAs from five randomly selected individuals and found non-designed mutations that were apparently induced by single or dual-sgRNAs of *AtMIR827a* (Supplementary Figure 4). These results indicate that a 6-bp deletion at the sgRNA1 target site occurred. At the same time, a 1-bp deletion was found in the right flanking side (RD1), indicating that the sgRNAs could act either individually or in combination to create different types of mutations, including mutations other than the mutations expected to be induced by either single sgRNA. However, it is relatively easy to screen for gene deletion and replacement mutations of interest using PCR methods. Another concern is off-target effects or mosaic mutations, which can be prevented by the use of a stable transformation cassette. *Arabidopsis* mutants can be mosaics in the T1 generation due to the constitutive overexpression of the CRISPR/Cas9 genome editing system^42^. To limit the constitutive mutagenesis activity and possible unexpected off-target effects produced by the CRISPR/Cas9 system, we screened for individuals that no longer contained the CRISPR/Cas9 expression cassette in the T1 generation. For a more refined analysis, possible off-target mutations could be further excluded by restoring the genetic background via backcrossing to WT plants.

## Materials and Methods

### Plant materials and genomic DNA extraction

*Arabidopsis thaliana* Col-0 ecotype was used as the WT control. Transformation and genome editing were also performed in the Col-0 WT genetic background. *Arabidopsis* seedlings germinated on Murashige-Skoog (MS) nutrient agar medium were transferred into soil and grown in a greenhouse under long-day conditions (16 h light at 23 °C and 8 h dark at 21 °C). The humidity was not controlled but was approximately 50%. Plant genomic DNA was extracted from the leaves using a TPS buffer plant DNA mini-prep protocol^43^.

### Construction of vectors

The human-optimized coding sequence of *SpCas9*^44^ was cloned into the CPB vector downstream of an enhanced CaMV 35S promoter. The *hSpCas9* region was amplified and cloned into the CPB vector using an In-Fusion® PCR Cloning Kit (Clontech, Mountain View, California, USA). The individual guide RNAs and their sequences are listed in Table 1. Two sgRNAs that directly target sequences located ~200 bp upstream and ~400 bp downstream of MIRNA169a were designed. These fragments were then cloned into the pCPB vector using the *Hind*III restriction site and an In-Fusion® HD Cloning Kit (Clontech, Mountain View, California, USA). The sequences of the target sites and the gRNA scaffold are shown in Tables 1 and 2. The *Arabidopsis thaliana* U6-26 promoter was used to drive the expression of the sgRNA genes. The promoters and the sgRNA genes were cloned into the CPB vector according to the manufacturer’s protocols. The Cas9 and dual-sgRNA expression cassette was constructed to form construct #1. Construct #1 was also modified for *MIR827a* deletion and *AtTFL1* replacement using different and specific dual-sgRNA sequences according to the same procedures. The detailed sequences of the key elements are presented in Supplementary File 10. The sequences and detailed information for all primers used in this study are provided in Supplementary File 11.

The vector of construct #2, which provided the donor (Fig. 1B) DNA repair template, was constructed and contained an enhanced 35S promoter driving the expression of eGFP with an SV 40 NLS (nuclear location signal sequence). The sequence details can be found in Supplementary File 12. The left and right homologous arms of *AtTFL1s* were constructed before and after the eGFP expression cassette. The 733-bp sequence was homologous to the left side of the target site in *AtTFL1* (see Supplementary File 10 &12), while the 825-bp sequence was homologous to the right side of the target site (see Supplementary File 10 &12). The expected residue sequenced after the excision of *AtTFL1* was also designed to contain the homologous arm (see Supplementary File 10).

### Transformation and identification of transformants

The plasmid was electroporated into *Agrobacterium tumefaciens* GV3301. For the single transformation of CRISPR/Cas9 and the co-transformation of both CRISPR/Cas9 and the DNA repair donors, a single clone of *Agrobacterium tumefaciens* GV3301 containing both vectors was confirmed by PCR. *Agrobacterium*-mediated *Arabidopsis* transformation was performed using the floral dip method, according to a reported method^45^. Transgenic plants were screened on MS agar plates containing 40 μM glufosinate ammonium. Transgenic plants were screened for gene replacement on MS agar plates containing 40 μM glufosinate ammonium and 15 μg/ml of hygromycin B. The glufosinate-resistant transformed plants were validated by PCR and with a strip test to detect the bialaphos resistance protein encoded by the *bar* gene (QuickStix™ Kit for LibertyLink® (*bar*), Portland, ME, USA).

### Mutant screening and identification

For screening large DNA deletion segments, PCR was performed with a pair of primers flanking the target sites. The primers were designed with Primer Premier 5.0, and the sequences are described below. The primer pair used for MIR169a mutation screening was Cas9-169 F: 5′-AGGATGGAGAAGCATGGAGG-3′ and Cas9-169 R: 5′-CTCATGGTTGGCAGCAGTTT-3′. The primer pair used for MIR827a mutation detection was Cas9-827 F: 5′-CCTTTTTTTCTGTAATCACCAGT-3′ and Cas9-827 R: 5′-AGCTTCAGAGGTTCCAAATACA-3′. The PCR amplicons were cloned into the PEASY-T1 vector (TransGen Biotech. Co. Catalog# CT101-02, Beijing, China). Single clones were picked for culture and sequencing. Sequencing reactions were performed using the M13-R primer (5′-CAGGAAACAGCTATGAC-3′). T1 or T2 heterozygous lines were screened and sequenced to confirm mutations.

The mutants (500 T0 individuals) were screened for *AtTFL1* replacement events via PCR using primer F1, which binds to the plant chromosome upstream or downstream of the targeted site (Fig. 1E and Supplementary File 1), in combination with the R2 primer, which binds to the targeted eGFP gene. Then, for identified replacement events, the entire sequence was amplified using primer pair F1 and R4 and confirmed by sequencing junction regions 1, 2, 3, and 4, and the full length of the desired gene was inserted into the target locus using the listed primers (Fig. 1E and Supplementary File 1).

The PCR amplicons were cloned into the pCR TA clone vector using a commercial kit (Transgene, Beijing China) based on the provided protocols and procedures. The M13 R primer was used for sequencing on an ABI3730 (Applied Biosystems, California, USA). The sequencing quality and results were viewed in Sequence Scanner Software ver2.0 (ABI Applied Biosystems) by importing raw sequencing trace files. The junction sites of the mutant were identified using the looking up tool in Sequence Scanner Software ver2.0. The sequencing peaks were shown by printing raw sequencing files in Adobe PDF format. The junction sites were located at the sequences shown in the different shadow colors.

### Screening for the targeting and deletion of the DNA donor template to facilitate HDR repair

Among the identified replacement-positive individuals in the T0 and T1 generations, the deletion of the DNA donor template was screened using primer pair F5 and R5. The original positive lines for target gene replacement were selected for additional experiments screening for DNA donor deletion. We selected T1 individuals in which the donor was clearly deleted. F5 and R5 were designed to include the *EcoR*I and *Hind*III restriction sites in both flanking regions outside of the dual-sgRNA/Cas9 target site (Fig. 1B, Fig. 4A), respectively. The sequence of F5 (BEcoRI-F) is 5′-TTGTGTGGAATTGTGAGCGG-3′. The sequence of R5 (AHindIII-R) is 5′-AAACTGAAGGCGGGAAACG-3′. The sequence details of the DNA repair donor and the expected sequence after target deletion are provided as Supplementary File 12.

### Microscopic screening of eGFP expression events and imaging

Identified positive replacement events in which the DNA donor was also identified as being clearly deleted were screened for eGFP expression. Mutants positive for both replacement and donor deletion were screened for eGFP expression using a 488-nm excitation wavelength on an MSHOT (Guangzhou, China) fluorescence microscope with 10 × 10 magnification. These eGFP-expressing plants were imaged to identify eGFP expression in both roots and leaves in living plants on a ZEISS (LSM700, Germany) microscope using a 488-nm excitation wavelength with 10 × 20 magnification.

### Drought tolerance phenotyping of mir169a

T3 homozygous lines were used for all phenotyping after verifying the mutations by DNA sequencing. To confirm the *mir169a* mutants, the drought response was phenotyped during the same treatments used in the original study that reported the functional identification of miR169a^24^. Briefly, the plants were grown in soil with sufficient water for 3 weeks before water was withheld for 12 days.

### RNA purification and miRNA northern blot analysis

Total RNA was extracted from WT and mutant plants using TRIzol® reagent (Invitrogen). For the enrichment of small RNAs, high-molecular weight RNA was selectively precipitated via the addition of 1 volume of 20% PEG/1 M NaCl^46^. Then, the remaining low-molecular weight RNA was fractionated on 17% denaturing polyacrylamide gels. The blots were probed and washed as described in the DIG Oligonucleotide Tailing Kit (Roche, City, USA). A conserved miRNA (miR172) was added as the loading control.

## Additional Information

**How to cite this article**: Zhao, Y. *et al*. An alternative strategy for targeted gene replacement in plants using a dual-sgRNA/Cas9 design. *Sci. Rep.* **6**, 23890; doi: 10.1038/srep23890 (2016).

## Supplementary Material

Supplementary Information

## Figures and Tables

**Table 1:** The targeted genes and design of the dual-sgRNA/Cas9-mediated targeted deletion to create null mutations via deletion or gene replacement.

Gene	Locus	sgRNA1 target sequence with PAM and the expected excision site (↓)	sgRNA2 target sequence with PAM and the expected excision site (↓)	Designed target deletion or replacement region
*AtMIR169a*	AT3G13405	5′-GAAATAGTTTCTAATTC↓TGGAGG-3′	5′-GAGATTTTATGCCCCCA↓AGAAGG-3′	Chr3:4558557..4559490 (–)
*AtMIR827a*	AT3G59884	5′-GGATCATCTATTGAAGG↓AACAGG-3′	5′-GCAAATCGAAAAGCTTC↓TTAGGG-3′	Chr3:22122674..22123280 (–)
*AtTFL1*	AT5G03840	5′-GCCATTGATAATGGGGA↓GAGTGG-3′	5′-GGAAAACTTGTAAGAGG↓AAAAGG-3′	Chr5:1025496..1025750 (–)*

Note: *The target region for gene replacement.

**Table 2:** The observed mutations of the deleted region along with inheritance and observed gene replacement events.

Gene	Deletion length (bp)	Amplicon length (WT/mutant) (bp)	Mutation observed no./total (T0) (frequency %)	Observed no. (T1)	Homozygote no. (T2)	Phenotypes verified no. (T3)
*AtMIR169a*	934	1132/206	10/50 (20.0%)	10	2	2
*AtMIR827a*	607	834/227	12/50 (24.0%)	11	4	4
*AtTFL1*	255	Sequencing	4/500 (0.8%)	4	3	NA

Note: *The homozygote lines were screened from 9 individuals of each T1 line. NA, not available.
