# Supplementary material for: An alternative strategy for targeted gene replacement in plants using a dual-sgRNA/Cas9 design
Source: Sci Rep. 2016 Apr 1;6:23890. doi: 10.1038/srep23890 (PMC4817149; doi:10.1038/srep23890)
Supplement: Supplementary Information [file srep23890-s1.pdf]

Supplementary materials information

Titile: An alternative strategy for targeted gene replacement in plants using a dual-sgRNA/Cas9 design

Authors: Yongping Zhao[1,#], Congsheng Zhang[1,2,#], Wenwen Liu[1], Wei Gao[1], Changlin Liu[1], Gaoyuan Song[1],Wen-Xue Li[1], Long Mao[1], Beijiu Chen[2], Yunbi Xu[1, 3], Xinhai Li[1], Chuanxiao Xie[1,]*

Author affiliations:

[1] Institute of Crop Science, Chinese Academy of Agricultural Sciences, National Key Facility for Crop Gene Resources and Genetic Improvement, Beijing, China 100081

[2] Anhui Agricultural University, Hefei, Anhui Province, China 230036

[3] International Maize and Wheat Improvement Center, El Batán, 56130, Mexico

I. The list of 5 supplementary figures.

Supplementary Figure 1. A scheme illustrating the designed targeted chromosome deletion region within the *AtTFL1* region within gene structure.

Empty rectangle, UTR region; black rectangle, exons; dash lines, introns; vertical arrows, the sgRNA CRISPR/Cas9 targeted sites.

Supplementary Figure 2. The design and sequence detail of dual-sgRNA CRISPR/Cas9 for deleting target region of *AtMIR169a* locus.

A. Expressed sgRNA1 targeting *AtMIR169a* at site 1. The PAM sequence AGG is underlined. PAM, protospacer-adjacent motif sequence (sequence NGG underlined in red.

B. Expressed sgRNA2 targeting *AtMIR169a* at site 2. The PAM sequence AGG is underlined. The sequences underlined in bold (A panel and B panel in this figure) are expected to be joined together after precision repair of both DSB lesions induced by the two sgRNAs.

Supplementary Figure 3. Screening of targeted deletion mutation lines of *mir169a* mutant in the T_1 generation.

M: DNA size marker (GeneRuler^{TM} 100-bp DNA Ladder; Fermentas, Beijing); WT, Columbia

Col0 wild type; 8, 12, 31, 32, 33, 34, 35, 36, 39, 42: the 10 lines in which the heterozygous targeted mutation was identified.

Supplementary Figure 4. The observed left (sgRNA1) or right (sgRNA2) indel mutations induced by single or dual sgRNAs rather than deletion of the entire target region in effort of deleting *AtMIR827* target region.

PAM, proto-adjacent motif; LD6, 6-bp deletion at the left sgRNA1-mediated site; RD1, 1-bp deletion at the right sgRNA2-mediated site. The number plus multiplication symbol indicates the observed individuals. For instance, for "3×" in figure panel B, 3 individuals had a 6-bp deletion mutation at the left sgRNA1 site (LD6) with no change at the sgRNA2 site.

Supplementary Figure 5. The sequencing evidences of the full length of the amplicon of the targeted replacement events. The full sequence could be seen in Supplementary File 1. The combined 4 junctions in one amplicon could identify a replacement event. Only junction sites 1-4 were shown. B: The junction site 1 sequence was shown; C: The junction site 2 was shown; D: The reverse complementary sequence of the junction 3. E: The reverse complementary sequence of the junction 4.

Note: Four raw sequencing file harboring 4 Junction sites had been provided as Supplementary File 2, 3, 4, and 5.

II. The list of 12 supplementary files.

Supplementary File 1. The desired sequence detail of partial AtTFL1 was replaced with eGFP.

Supplementary File 2. The raw sequencing peaks surrounding the junction 1 of the replacement event.

Supplementary File 3. The raw sequencing peaks surrounding the junction 2 of the replacement event.

Supplementary File 4. The raw sequencing peaks of the reverse complementary sequence surrounding the junction 3 of the replacement event.

Supplementary File 5. The raw sequencing peaks of the reverse complementary sequence surrounding the junction 4 of the replacement event.

Supplementary File 6. The raw sequencing peak of the reverse complementary sequence harboring re-joining junction site after DNA donor had been target deleted in plant.

Supplementary File 7, 8 and 9. Three more biological replicates of the sequencing evidence for harboring re-joining junction site after DNA donor had been target deleted in plants.

Supplementary File 10. The sequence of the mentioned key elements of CRISPR/Cas9 expression cassette in this study.

Supplementary File 11. The list of primers used in this study.

Supplementary File 12. The sequence detail of DNA donor template before and after deletion to supply for HDR repair.

Title：An alternative strategy for targeted gene replacement in plants using a dual-sgRNA/Cas9 design
Authors： Zhao Y., Zhang C., Liu W., Gao W., Liu C., Song G., Li W-X, Mao L., Chen B., Xu Y., Li X., Xie C.

Supplementary Figure 1

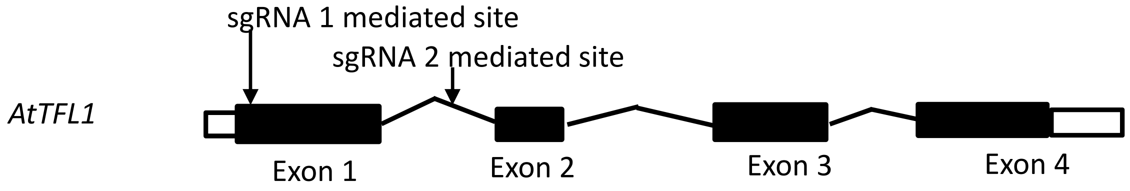

Supplementary Figure 1. A scheme illustrating the designed targeted chromosome deletion region within the *AtTFL1* region within gene structure. Empty rectangle, UTR region; black rectangle, exons; dash lines, introns; vertical arrows, the sgRNA CRISPR/Cas9 targeted sites.

Title： An alternative strategy for targeted gene replacement in plants using a dual-sgRNA/Cas9 design

Authors： Zhao Y., Zhang C., Liu W., Gao W., Liu C., Song G., Li W-X, Mao L., Chen B., Xu Y., Li X., Xie C.

Supplementary Figure 2

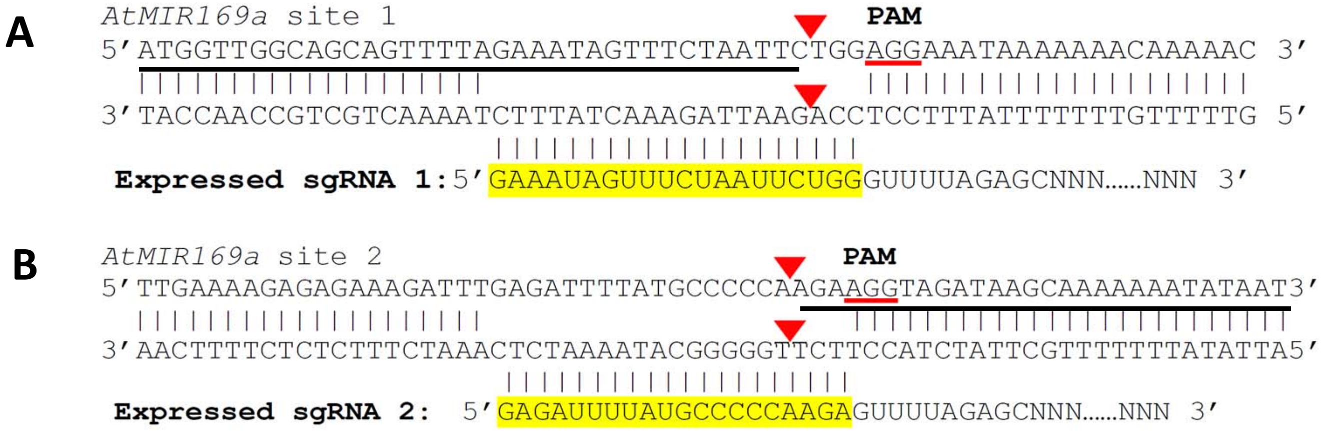

Supplementary Figure 2. The design and sequence detail of dual-sgRNA CRISPR/Cas9 for deleting target region of *AtMIR169a* locus.

A. Expressed sgRNA1 targeting *AtMIR169a* at site 1. The PAM sequence AGG is underlined. PAM, protospacer-adjacent motif sequence (sequence NGG underlined in red.

B. Expressed sgRNA2 targeting *AtMIR169a* at site 2. The PAM sequence AGG is underlined. The sequences underlined in bold (A panel and B panel in this figure) are expected to be joined together after precision repair of both DSB lesions induced by the two sgRNAs.

Title: An alternative strategy for targeted gene replacement in plants using a dual-sgRNA/Cas9 design

Authors: Zhao Y., Zhang C., Liu W., Gao W., Liu C., Song G., Li W-X, Mao L., Chen B., Xu Y., Li X., Xie C.

Supplementary Figure 3

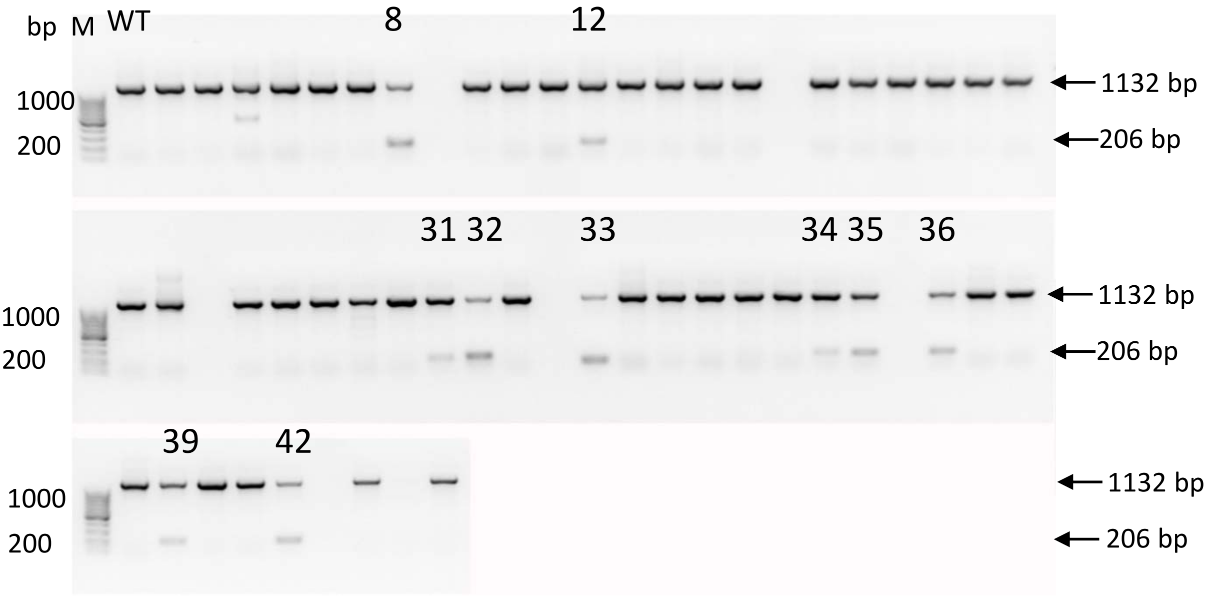

Supplementary Figure 3. Screening of targeted deletion mutation lines of *mir169a* mutant in the T_1 generation.
M: DNA size marker (GeneRuler^{TM} 100-bp DNA Ladder; Fermentas, Beijing); WT, Columbia Col0 wild type; 8, 12, 31, 32, 33, 34, 35, 36, 39, 42: the 10 lines in which the heterozygous targeted mutation was identified.

Title: An alternative strategy for targeted gene replacement in plants using a dual-sgRNA/Cas9 design

Authors: Zhao Y., Zhang C., Liu W., Gao W., Liu C., Song G., Li W-X, Mao L., Chen B., Xu Y., Li X., Xie C.

Supplementary Figure 4

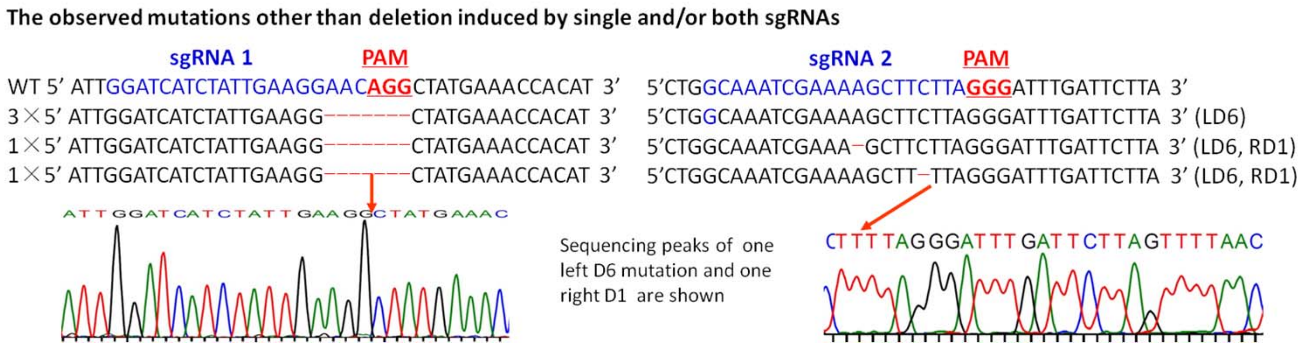

Supplementary Figure 4. The observed left (sgRNA1) or right (sgRNA2) indel mutations induced by single or dual sgRNAs rather than deletion of the entire target region in effort of deleting *AtMIR827* target region.

PAM, proto-adjacent motif; LD6, 6-bp deletion at the left sgRNA1-mediated site; RD1, 1-bp deletion at the right sgRNA2-mediated site. The number plus multiplication symbol indicates the observed individuals. For instance, for "3×" in figure panel B, 3 individuals had a 6-bp deletion mutation at the left sgRNA1 site (LD6) with no change at the sgRNA2 site.

Title: An alternative strategy for targeted gene replacement in plants using a dual-sgRNA/Cas9 design

Authors: Zhao Y., Zhang C., Liu W., Gao W., Liu C., Song G., Li W-X, Mao L., Chen B., Xu Y., Li X., Xie C.

Supplementary Figure 5

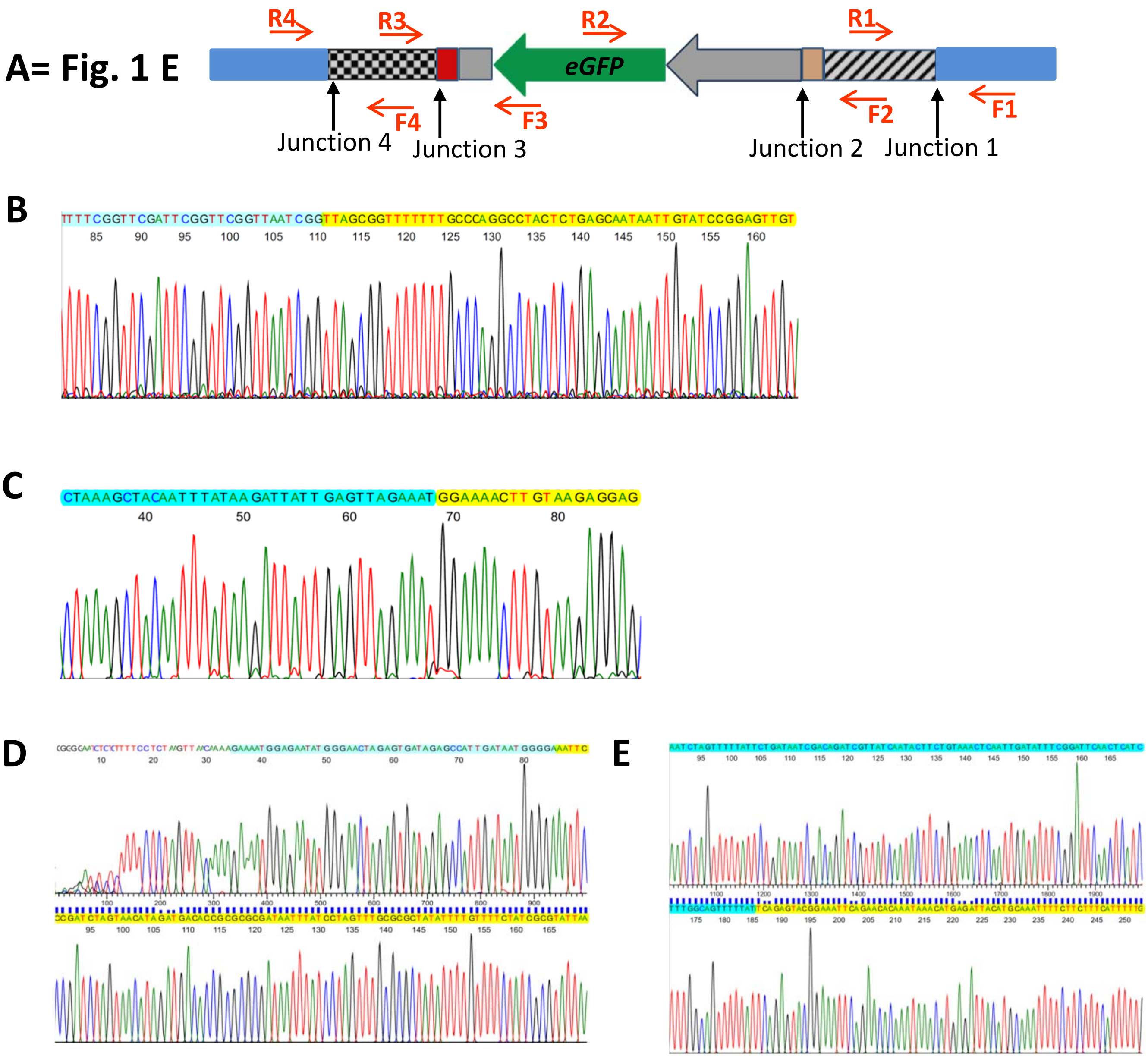

Supplementary Figure 5. The sequencing evidences of the full length of the amplicon of the targeted replacement events. The full sequence could be seen in Supplementary File 1. The combined 4 junctions in one amplicon could identify a replacement event. Only junction sites 1-4 were shown. B: The junction site 1 sequence was shown; C: The junction site 2 was shown; D: The reverse complementary sequence of the junction 3. E: The reverse complementary sequence of the junction 4.

Note: Four raw sequencing file harboring 4 Junction sites had been provided as Supplementary File 2, 3, 4, and 5.

Title：An alternative strategy for targeted gene replacement in plants using a dual-sgRNA/Cas9 design

Authors： Zhao Y., Zhang C., Liu W., Gao W., Liu C., Song G., Li W-X, Mao L., Chen B., Xu Y., Li X., Xie C.

Supplementary file 1. The desired sequence detail of partial AtTFL1 was replaced with eGFP.

This sequence was confirmed with assembled sequencing from 1 amplicon using primer pair of F1 and R4.

```
                F1 primer
1      ctttggtttt catttggtta tcgGTTATTA ACCTAACCGA AACCGAAACC GAAATCTAAG
61     ACATATAATA TTCAACCGGT TATTTTAAGC TATCCAAACC TGAACCGAAC CATGTTTTTC
                              Junction 1
121    GGTTCGATTC GGTTCGGTTA ATCGGTTAGC GGTTTTTTTG CCCAGGCCTA CTCTGAGCAA
181    TAATTGTATC CGGAGTTGTA ATAGAATCAA AGTACGATGA GAGTGTTTTT ATGACAAATA
241    TCTTAATCTT GGCCAATTAT ATGTTCTACT GAAATTCTTT TTGAATTCAT CGACCAGTGA
301    GACTTAAAAA TAGCTTTTTA TTCGCCGAGG TATATATAGC TAGGAATTTT GTCGAAATTT
361    AGACGTTAGT GGGTTTTGTT CTTCGTGACA CAAAAGATAT TCTATATATT AACGAAATCT
421    AGCGATCGAT ATGGTATTTA TATAAAGTCT TGGTCATAGA TAGGGGTTGA AACTTGAAAC
481    CATGCATGAT ATGCCAATGT TGCTGAAGCA GTCAATGTTG CTGAAGAAGT CAAACGTAAT
541    TATATAGTGA ATACCAAAAA AGTGATATTT CTTAATTCAA TTAAATATAA TTATAGTTTT
601    AAATCACCTA AAATAAGTTA CTTATTAAAA CCCCCCAAAT TTACTTTAAT ATAGTTGGTG
661    TACATGTTTG AGAAAGCAAA CAAAAAGAAA AAGAAAAAGA AAAAAAAAAG AGAAAGAGGT
721    TAGTACACAT AATTGGGAAT TAATGTCTAT TGATTCTTTT ATCTTTCTCT CTCTCTCTAA
                                 F2 and R1 primer site
781    GACGGAAAAC CCCTATAAAT AGatgtctcg gtcgtctctt tctctCCCAA ATCACTACAA
                                                start codon of AtTFL1
841    ATCTCTCTTT TCCTCTAAGT TAACAAAAGA AAATGGAGAA TATGGGAACT AGAGTGATAG
          sgRNA1 targeted residue and Junction 2
901    AGCCATTGAT AATGGGGAAA TTCCCGATCT AGTAACATAG ATGACACCGC GCGCGATAAT
961    TTATCCTAGT TTGCGCGCTA TATTTTGTTT TCTATCGCGT ATTAAATGTA TAATTGCGGG
1021   ACTCTAATCA TAAAAACCCA TCTCATAAAT AACGTCATGC ATTACATGTT AATTATTACA
1081   TGCTTAACGT AATTCAACAG AAATTATATG ATAATCATCG CAAGACCGGC AACAGGATTC
1141   AATCTTAAGA AACTTTATTG CCAAATGTTT GAACGATCGG GGAAATTCGA GCTCGGTACC
                     SV40  NLS
1201   CGGGCGATCA TACCTTTCTC TTCTTCTTGG GAGAACCCCC TTTGTACAGC TCGTCCATGC
1261   CGTGAGTGAT CCCGGCGGCG GTCACGAACT CCAGCAGGAC CATGTGATCG CGCTTCTCGT
1321   TGGGGTCTTT GCTCAGGGCG GACTGGGTGC TCAGGTAGTG GTTGTCGGGC AGCAGCACGG
1381   GGCCGTCGCC GATGGGGGTG TTCTGCTGGT AGTGGTCGGC GAGCTGCACG CTGCCGTCCT
1441   CGATGTTGTG GCGGATCTTG AAGTTCACCT TGATGCCGTT CTTCTGCTTG TCGGCCATGA
1501   TATAGACGTT GTGGCTGTTG TAGTTGTACT CCAGCTTGTG CCCCAGGATG TTGCCGTCCT
1561   CCTTGAAGTC GATGCCCTTC AGCTCGATGC GGTTCACCAG GGTGTCGCCC TCGAACTTCA
1621   CCTCGGCGCG GGTCTTGTAG TTGCCGTCGT CCTTGAAGAA GATGGTGCGC TCCTGGACGT
1681   AGCCTTCGGG CATGGCGGAC TTGAAGAAGT CGTGCTGCTT CATGTGGTCG GGGTAGCGGC
1741   TGAAGCACTG CACGCCGTAG GTGAAGGTGG TCACGAGGGT GGGCCAGGGC ACGGGCAGCT
1801   TGCCGGTGGT gcagatgaacttcagggtca GCTTGCCGTA GGTGGCATCG CCCTCGCCCT
1861   CGCCGGACAC GCTGAACTTG TGGCCGTTTA CGTCGCCGTC CAGCTCGACC AGGATGGGCA
                              R2 primer (in GFP)
1921   CCACCCCGGT GAACAGctcc tcgcccttgc tcaccatCCC GGGGATCCTC TAGAGTCCCC
1981   CGTGTTCTCT CCAAATGAAA TGAACTTCCT TATATAGAGG AAGGGTCTTG CGAAGGATAG
```

2041 TGGGATTGTG CGTCATCCCT TACGTCAGTG GAGATATCAC ATCAATCCAC TTGCTTTGAA
2101 GACGTGGTTG GAACGTCTTC TTTTTCCACG ATGCTCCTCG TGGGTGGGGG TCCATCTTTG
2161 GGACCACTGT CGgcagaggc atcttcaacg atGGCCTTTC CTTTATCGCA ATGATGGCAT

F3 primer region

2221 TTGTAGGAGC CACCTTCCTT TTCCACTATC TTCACAATAA AGTGACAGAT AGCTGGGCAA
2281 TGGAATCCGA GGAGGTTTCC GGATATTACC CTTTGTTGAA AAGTCTCAAT TGCCCTTTGG
2341 TCTTCTGAGA CTGTATCTTT GATATTTTTG GAGTAGACAA GTGTGTCGTG CTCCACCATG
2401 TTGACGAAGA TTTTCTTCTT GTCATTGAGT CGTAAGAGAC TCTGTATGAA CTGTTCGCCA
2461 GTCTTTACGG CGAGTTCTGT TAGGTCCTCT ATTTGAATCT TTGACTCCAT GGCCTTTGAT
2521 TCAGTGGGAA CTACCTTTTT AGAGACTCCA ATCTCTATTA CTTGCCTTGG TTTGTGAAGC
2581 AAGCCTTGAA TCGTCCATAC TGGAATAGTA CTTCTGATCT TGAGAAATAT ATCTTTCTCT
2641 GTGTTCTTGA TGCAGTTAGT CCTGAATCTT TTGACTGCAT CTTTAACCTT CTTGGGAAGG
2701 TATTTGATTT CCTGGAGATT ATTGCTCGGG TAGATCGTCT TGATGAGACC TGCTGCGTAA
2761 GCCTCTCTAA CCATCTGTGG GTTAGCATTC TTTCTGAAAT TGAAAAGGCT AATCTGGGGA

Junction 3 and the sgRNA2 targeted residue were bold letters

2821 CCTGCAGGCA TGCAAGCT**CC TCTTACAAGT TTT**CCATTTC TAACTCAATA ATCTTATAAA
2881 TTGTAGCTTT AGTTTTTATC ATTCCTTTTT CCAGTCTTTT TTTTTTAATG GTAAAACTCA

R3 and F4 primers region

2941 ACcgaaatgc aaaacaggtc atgATAGACC CAGATGTTCC AGGTCCTAGT GACCCCTTTC
3001 TAAAAGAACA CCTGCACTGG TACGTTTAAT TTATTTATTC TTTCTTTTCA TTTTGGGCCC
3061 ATATTCCATA TACATTGCAT TTAAATCATT TCGTTATAAC CCTAATAAAG TTTTTTTTGG
3121 GTGTAAGTTA TATACATTTG AGTTGGTCAA AGATCTCCAT CGCCATGAGT TCTCAGAACT
3181 TTTTCTGTAA AGTAATAATA TTAGTATTGT TGAATGTTTC AATAGGATCG TTACAAACAT
3241 TCCCGGCACA ACAGATGCTA CGTTTGGTAA GGCCTCTTCA TGAATCTTGT AATTTAAATA
3301 CTTATACATA TATCATGTTA TATAGAAATA AAAATATTTG CATTGTAATA TAGGCAAAGA
3361 GGTGGTGAGC TATGAATTGC CAAGGCCAAG CATAGGGATA CATAGGTTTG TGTTTGTTCT
3421 GTTCAGGCAG AAGCAAAGAC GTGTTATCTT TCCTAATATC CCTTCGAGAG ATCACTTCAA
3481 CACTCGTAAA TTTGCGGTCG AGTATGATCT TGGTCTCCCT GTCGCGGCCG TCTTCTTTAA
3541 CGCACAAAGA GAAACCGCTG CACGCAAACG CTAGTTTCAT GATTGTCATA AACTGCAAAA
3601 ATGAAAGAAG AAAATTTGCA TGTAATCTCA TGTTTATTTG TGTTCTGAAT TTCCGTACTC
3661 TGAATAAAAA CTGCCAAAGA TGAGTTGAAT CCGAAATATC AATTGAGTTT ACAGAAGTAT

Junction4

3721 TGATAACGAT CTGTCGATTA TCAGAATAAA AACTAGATTA ATTGCATATC ATGTTTAGCA
3781 TTGTAATACT ACAAAAATAG TAAACTCTTG ATTAATTAAT AAAATCTAAG TTGCTGTAGT
3841 ATATAAATCA TTAAATCctc atacatggct tgataggtca c

R4 primer region

Captions:

- Sequence in red letters is the replaced sequence harboring the expression cassette of eGFP.
- Sequence with light blue shadowed is the sequence of the homologs arms.
- All primer regions were underlined in lowercase letters.
- At the middle junction sites.
- The captions were inserted between the lines of the sequence.
- The junction sites 1-4 were indicated. The different color letters in word "junction" indicate the real junction site located in the sequence. For example, "Junction 1" indicates that the junction sites were located within "ATCGGTTAGC" showing different shadow colors. "GT" was the very junction site.

Title ： An alternative strategy for targeted gene replacement in plants using a dual-sgRNA/Cas9 design

Authors： Zhao Y., Zhang C., Liu W., Gao W., Liu C., Song G., Li W-X, Mao L., Chen B., Xu Y., Li X., Xie C.

Supplementary File 2. The raw sequencing peaks surrounding the junction 1 of the replacement event.

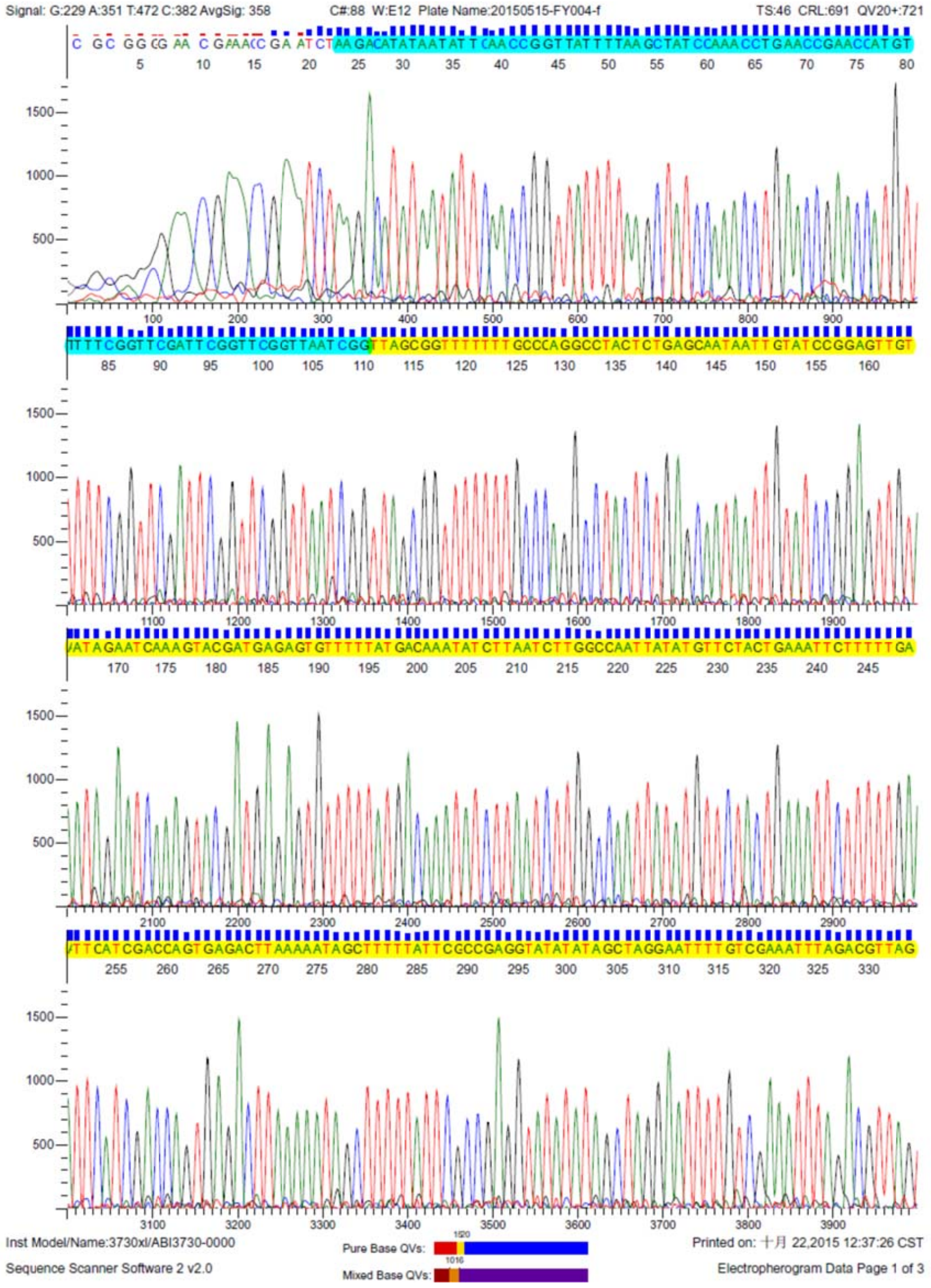

Signal: G:229 A:351 T:472 C:382 AvgSig: 358 C#:88 W:E12 Plate Name:20150515-FY004-f TS:46 CRL:691 QV20+:721

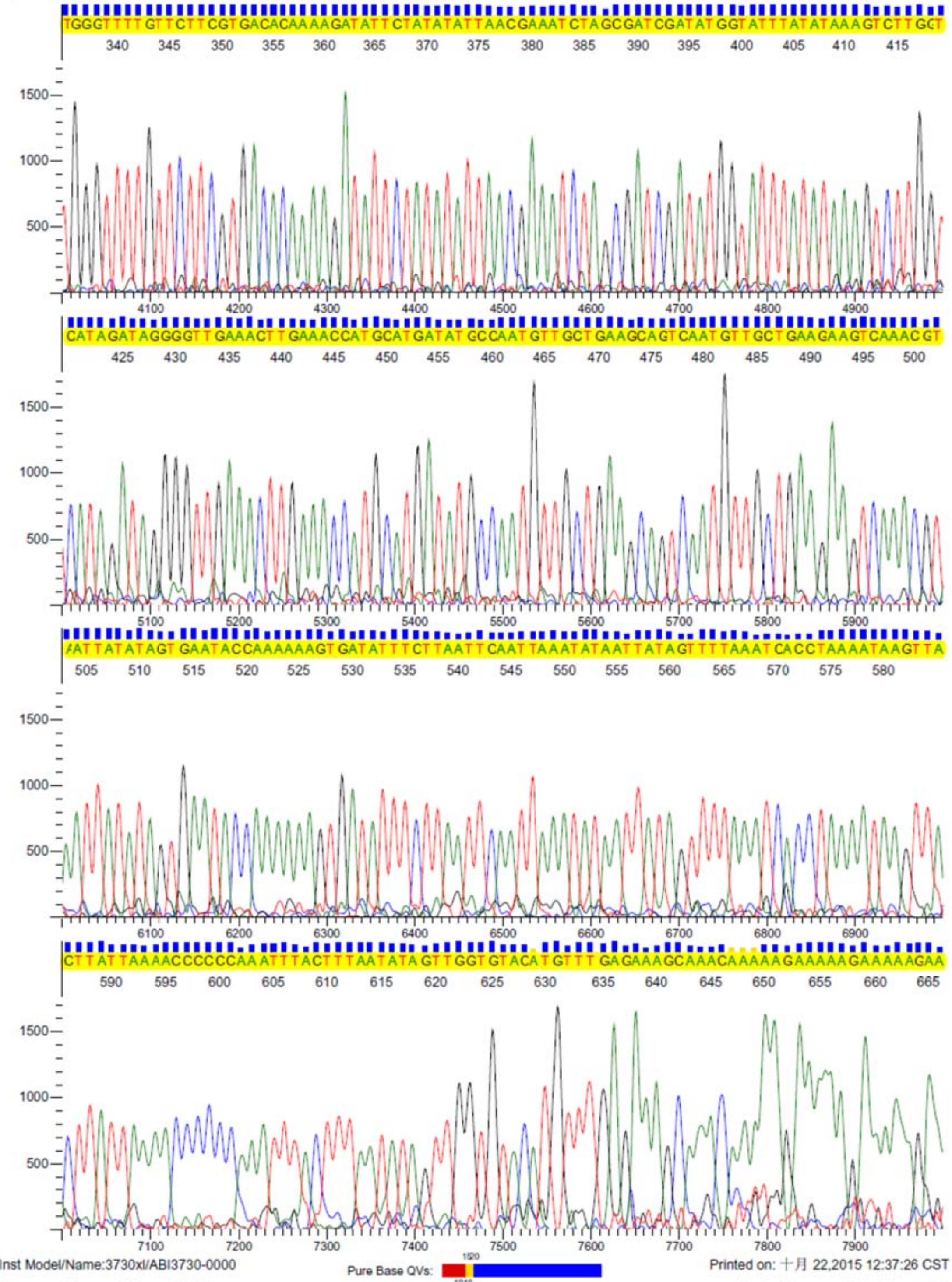

Inst Model/Name:3730xl/ABI3730-0000

Pure Base QVs:

Mixed Base QVs:

Printed on: 十月 22,2015 12:37:26 CST

Sequence Scanner Software 2 v2.0

Title : An alternative strategy for targeted gene replacement in plants using a dual-sgRNA/Cas9 design

Authors: Zhao Y., Zhang C., Liu W., Gao W., Liu C., Song G., Li W-X, Mao L., Chen B., Xu Y., Li X., Xie C.

Supplementary File 3. The raw sequencing peaks surrounding the junction 2 of the replacement event.

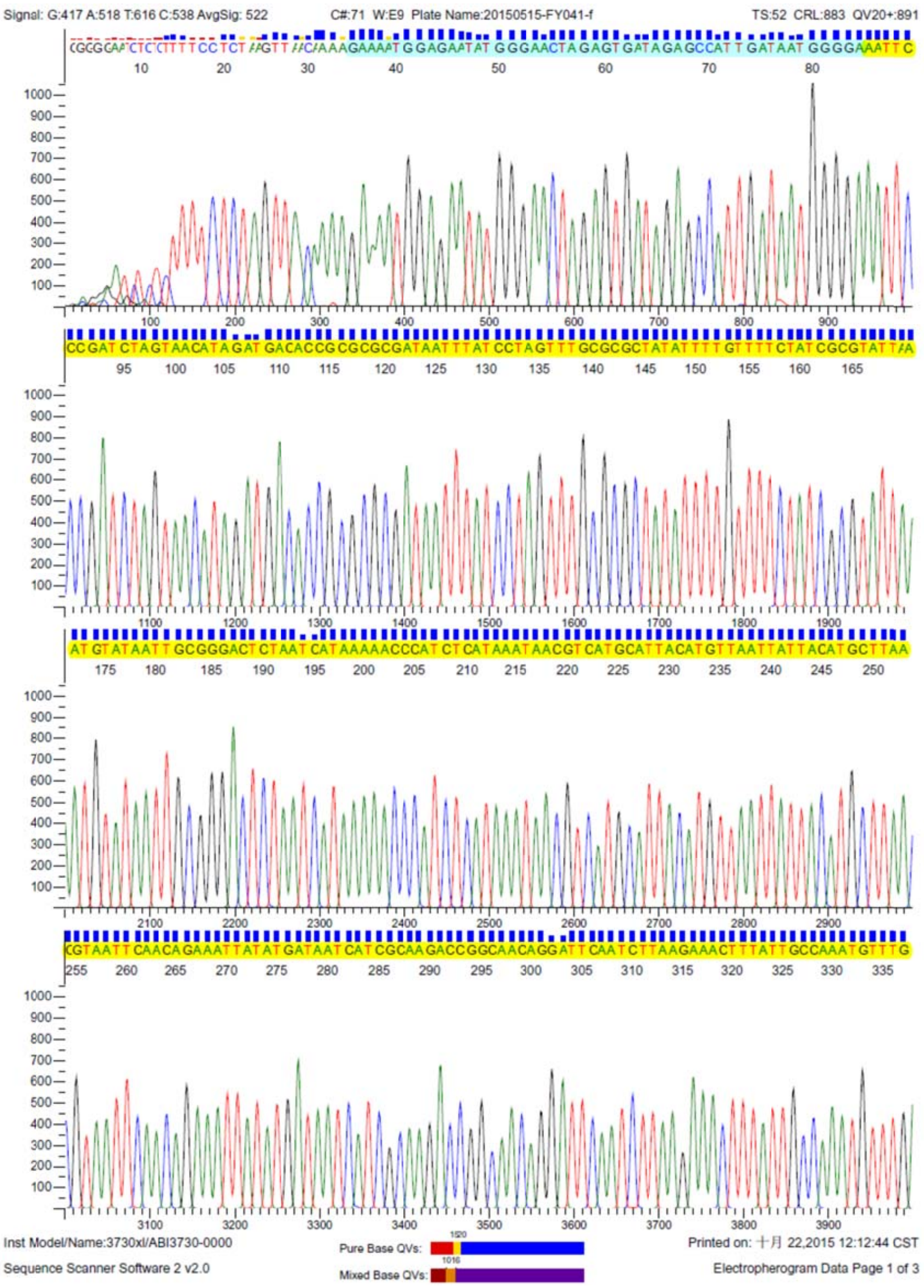

Signal: G:417 A:518 T:616 C:538 AvgSig: 522 C#:71 W:E9 Plate Name:20150515-FY041-f TS:52 CRL:883 QV20+:891

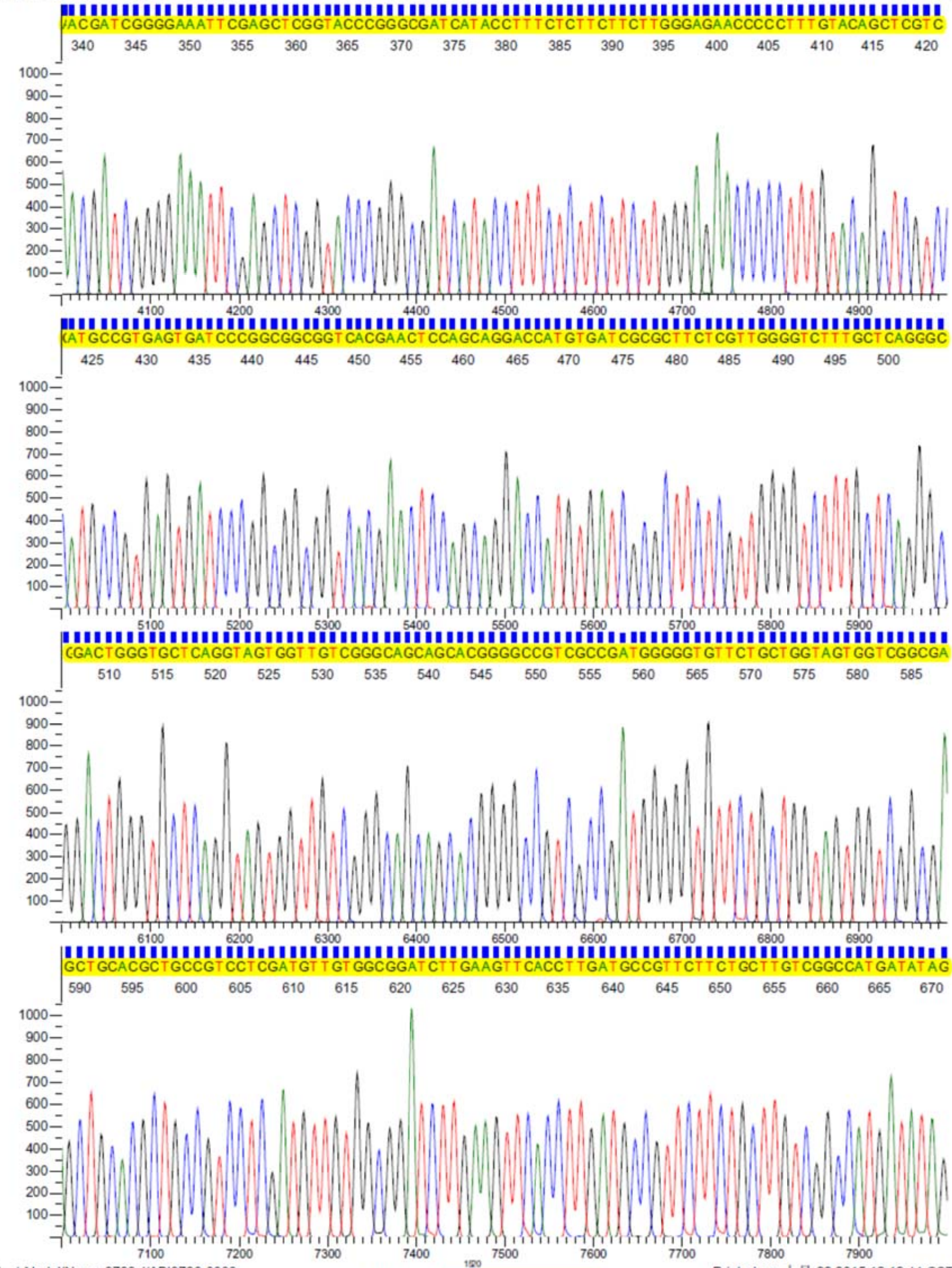

Inst Model/Name:3730xl/ABI3730-0000 Pure Base QVs: Printed on: 十月 22,2015 12:12:44 CST

Sequence Scanner Software 2 v2.0 Mixed Base QVs: Electropherogram Data Page 2 of 3

Signal: G:417 A:518 T:616 C:538 AvgSig: 522 C#:71 W:E9 Plate Name:20150515-FY041-f TS:52 CRL:883 QV20+:891

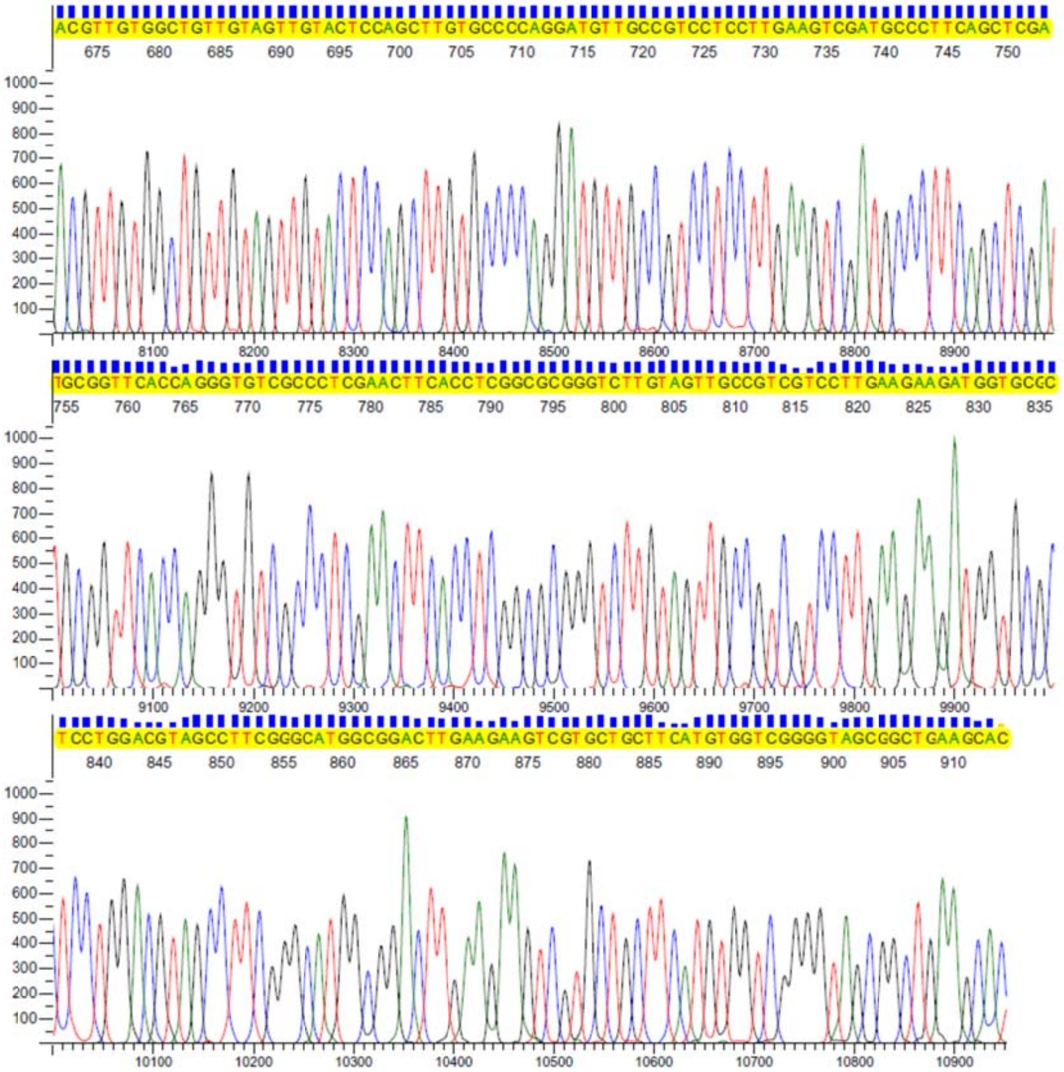

Inst Model/Name:3730xl/ABI3730-0000

Sequence Scanner Software 2 v2.0

Pure Base QVs:

Mixed Base QVs:

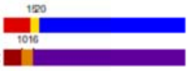

Printed on: 十月 22,2015 12:12:44 CST

Title : An alternative strategy for targeted gene replacement in plants using a dual-sgRNA/Cas9 design

Authors: Zhao Y., Zhang C., Liu W., Gao W., Liu C., Song G., Li W-X, Mao L., Chen B., Xu Y., Li X., Xie C.

Supplementary File 4. The raw sequencing peaks of the reverse complementary sequence surrounding the junction 3 of the replacement event.

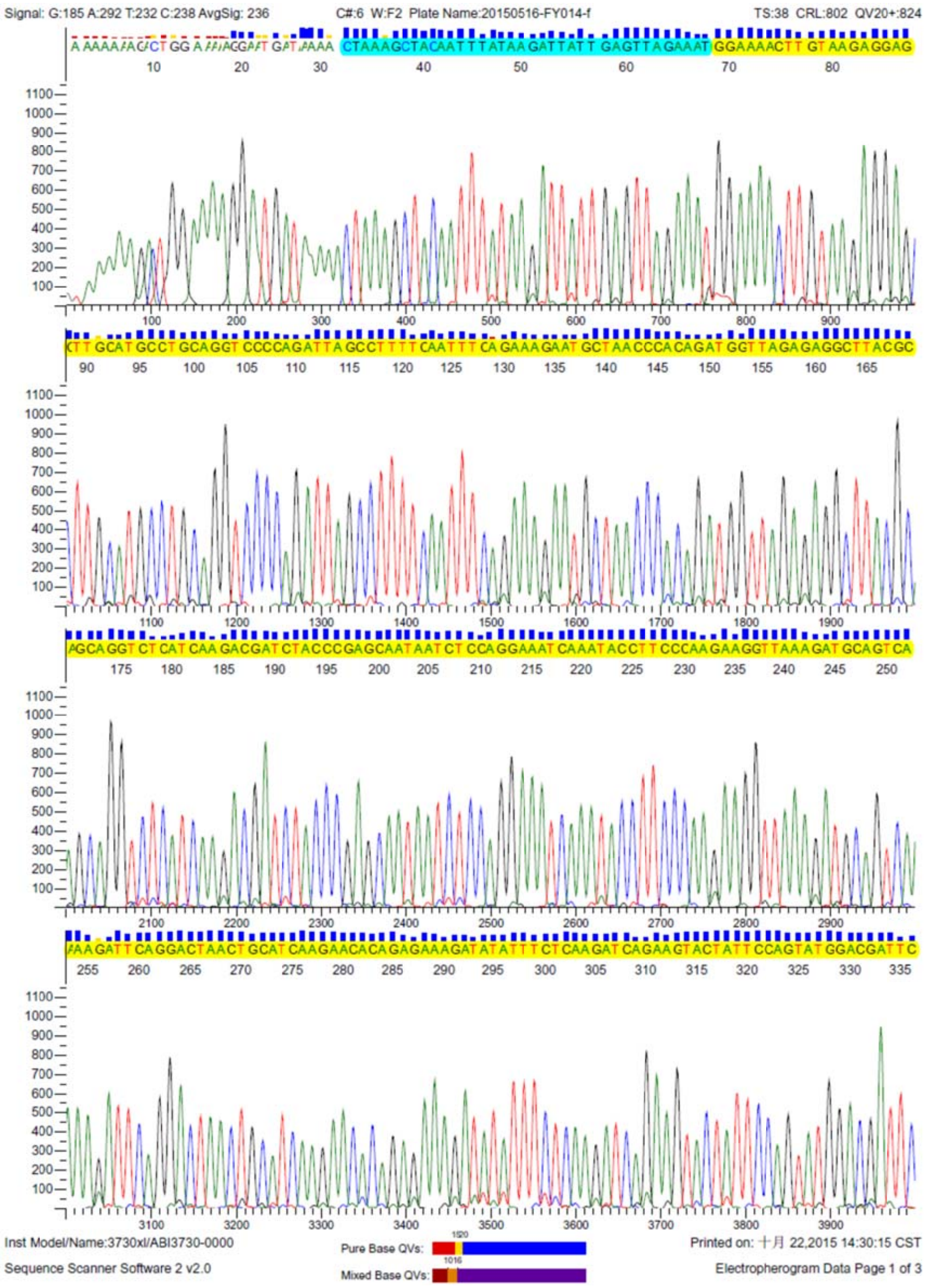

Signal: G:185 A:292 T:232 C:238 AvgSig: 236 C#:6 W:F2 Plate Name:20150516-FY014-f TS:38 CRL:802 QV20+:824

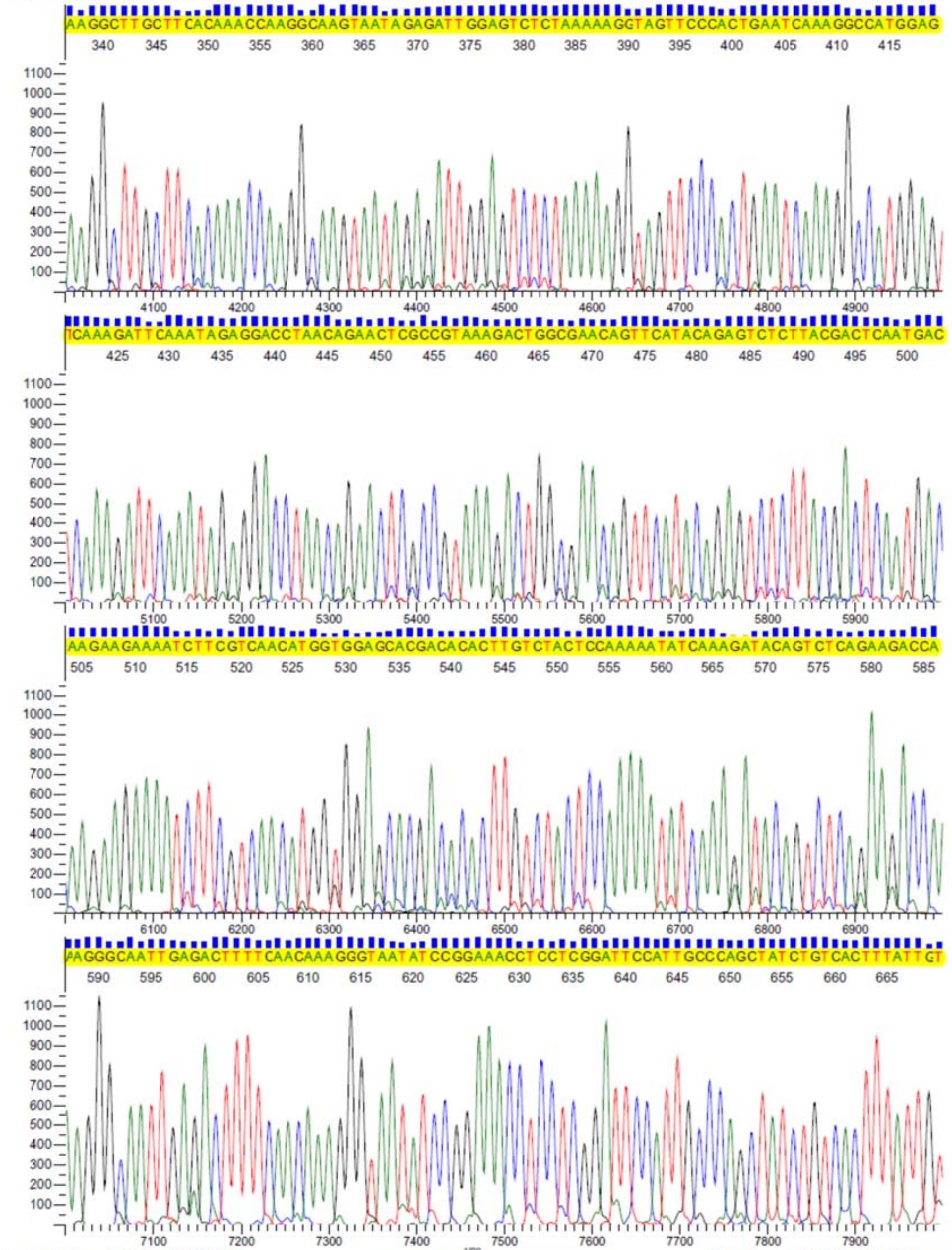

Inst Model/Name:3730xl/ABI3730-0000

Pure Base QVs: 1520

Mixed Base QVs: 1016

Printed on: 十月 22,2015 14:30:15 CST

Sequence Scanner Software 2 v2.0

Signal: G:185 A:292 T:232 C:238 AvgSig: 236 C#:6 W:F2 Plate Name:20150516-FY014-f TS:38 CRL:802 QV20+:824

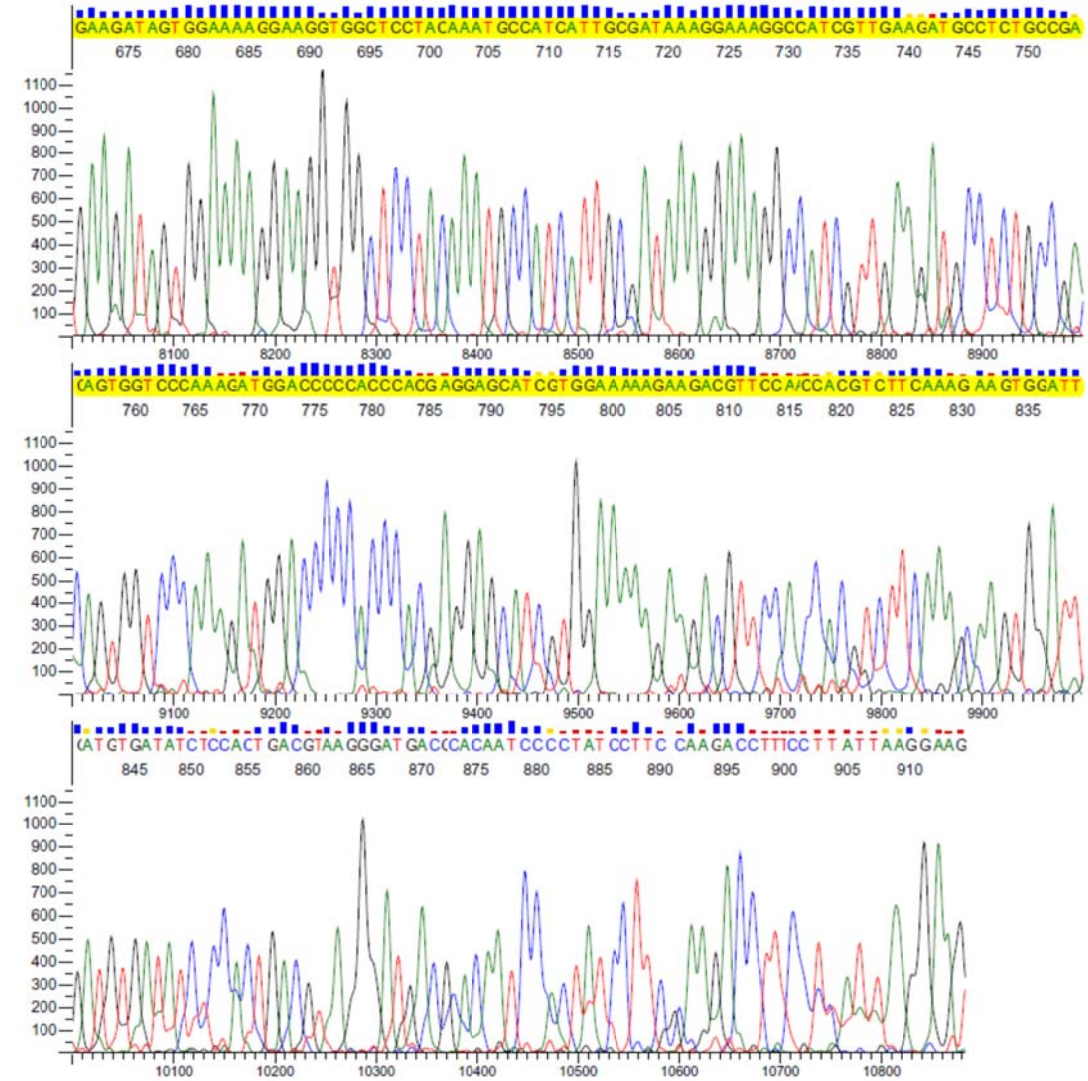

Title : An alternative strategy for targeted gene replacement in plants using a dual-sgRNA/Cas9 design

Authors: Zhao Y., Zhang C., Liu W., Gao W., Liu C., Song G., Li W-X, Mao L., Chen B., Xu Y., Li X., Xie C.

Supplementary File 5. The raw sequencing peaks of the reverse complementary sequence surrounding the junction 4 of the replacement event.

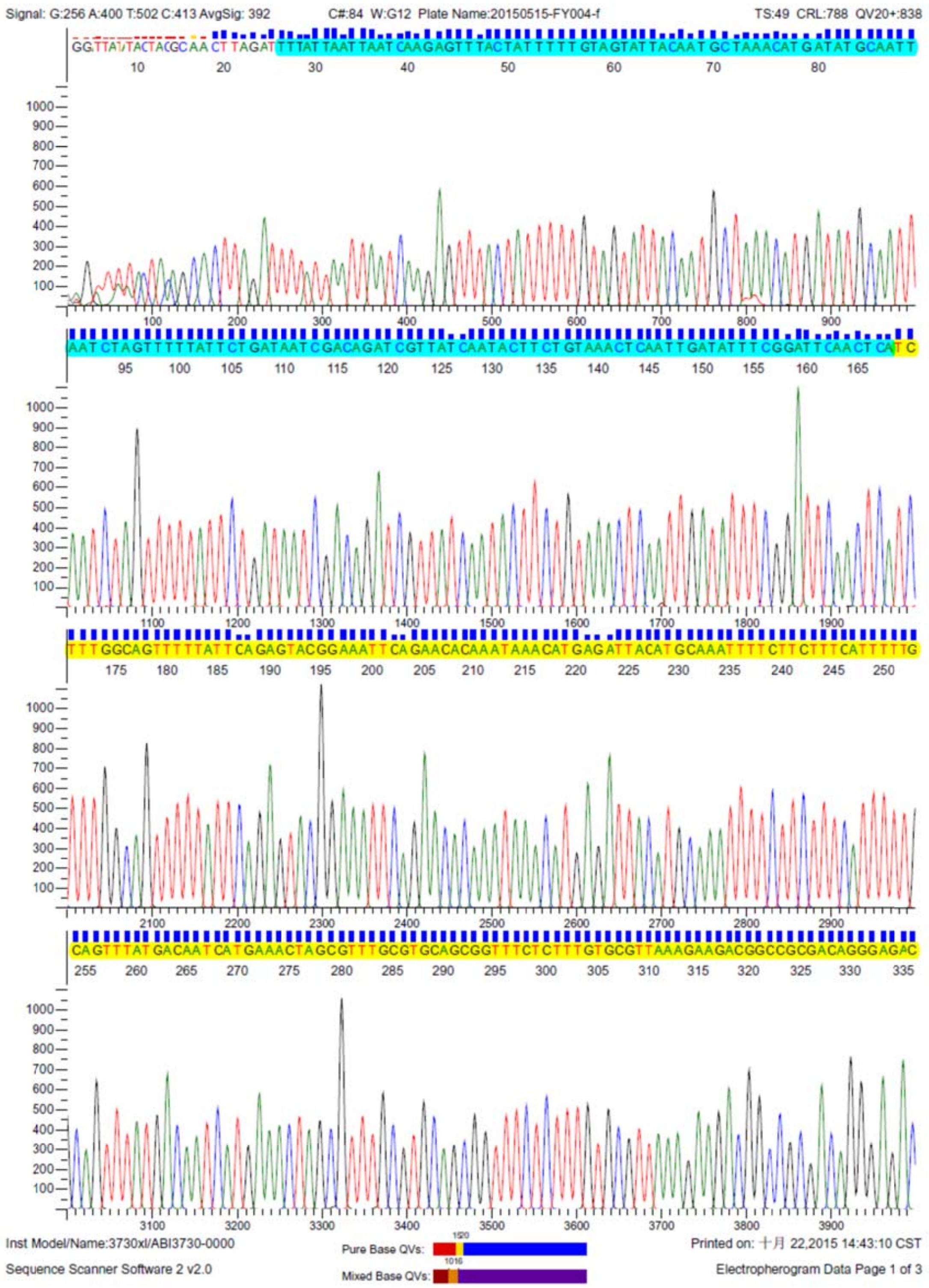

Signal: G:256 A:400 T:502 C:413 AvgSig: 392 C#:84 W:G12 Plate Name:20150515-FY004-f TS:49 CRL:788 QV20+:838

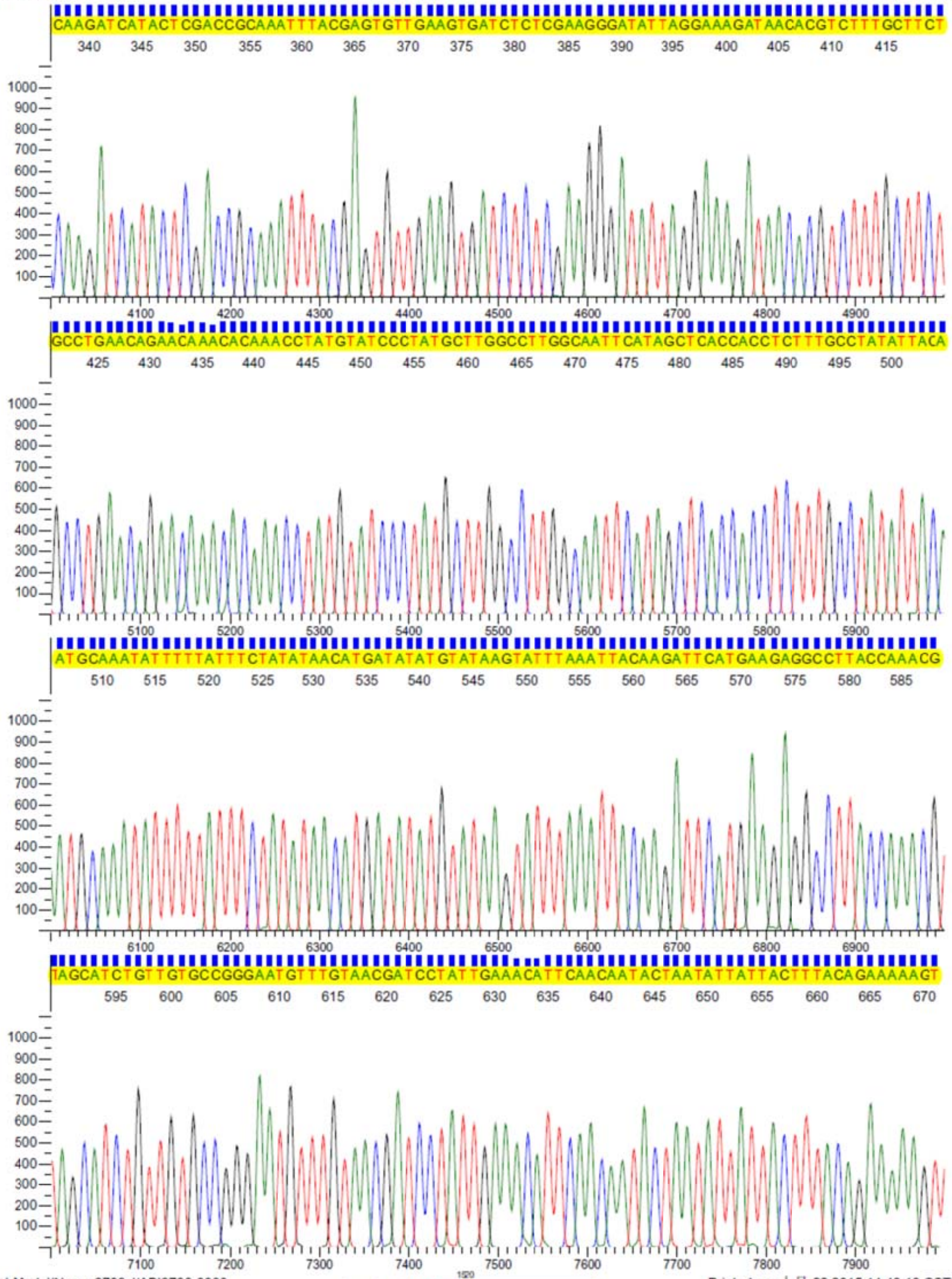

Inst Model/Name:3730xl/ABI3730-0000 Pure Base QVs: Printed on: 十月 22,2015 14:43:10 CST

Sequence Scanner Software 2 v2.0 Mixed Base QVs:

Signal: G:256 A:400 T:502 C:413 AvgSig: 392   C#:84 W:G12 Plate Name:20150515-FY004-f   TS:49 CRL:788 QV20+:838

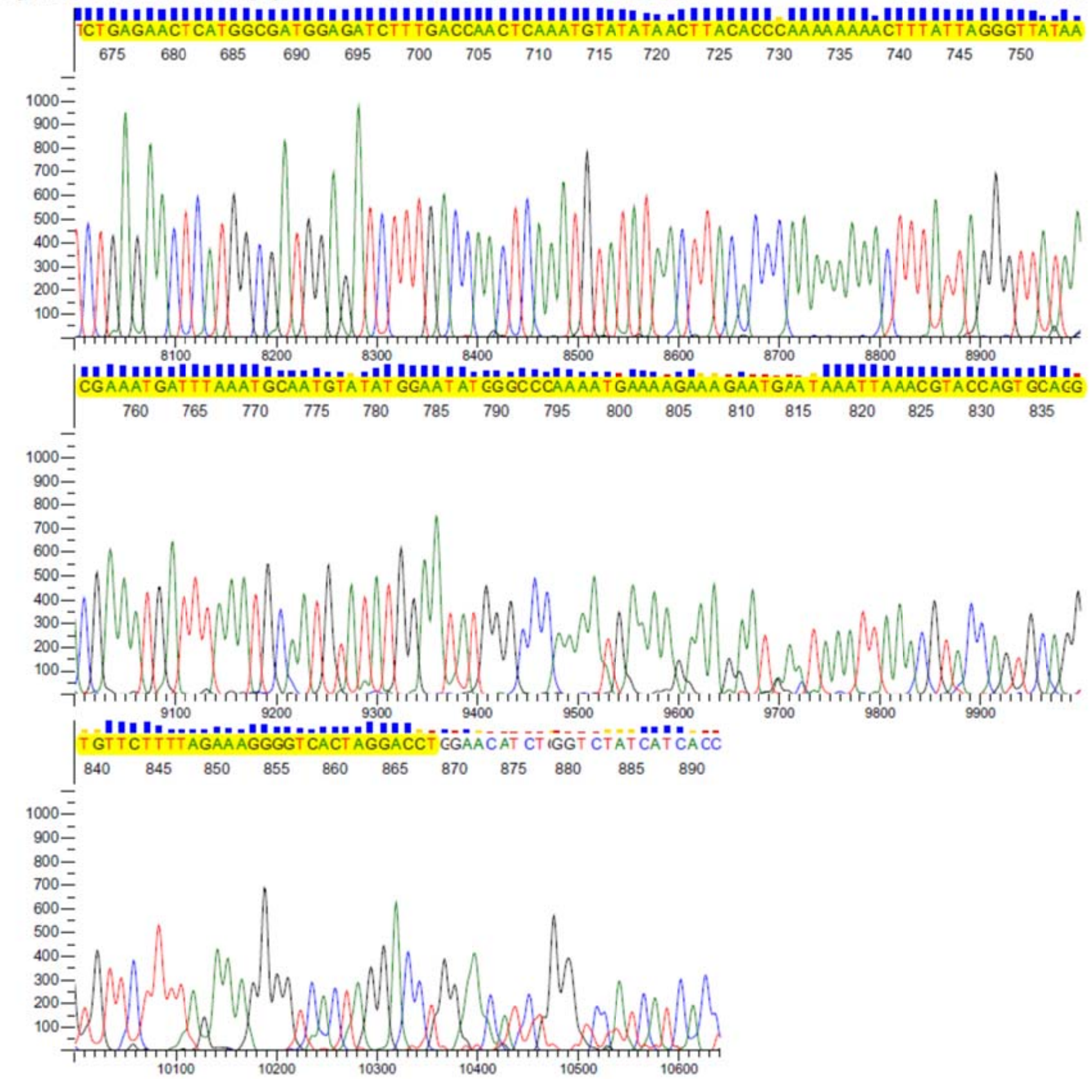

Inst Model/Name:3730xl/ABI3730-0000   Pure Base QVs: 1520   Printed on: 十月 22,2015 14:43:10 CST

Sequence Scanner Software 2 v2.0   Mixed Base QVs: 1016

Title : An alternative strategy for targeted gene replacement in plants using a dual-sgRNA/Cas9 design

Authors: Zhao Y., Zhang C., Liu W., Gao W., Liu C., Song G., Li W-X, Mao L., Chen B., Xu Y., Li X., Xie C.

Supplementary File 6. The raw sequencing peak of the reverse complementary sequence harboring re-joining junction site after DNA donor had been target deleted in plant.

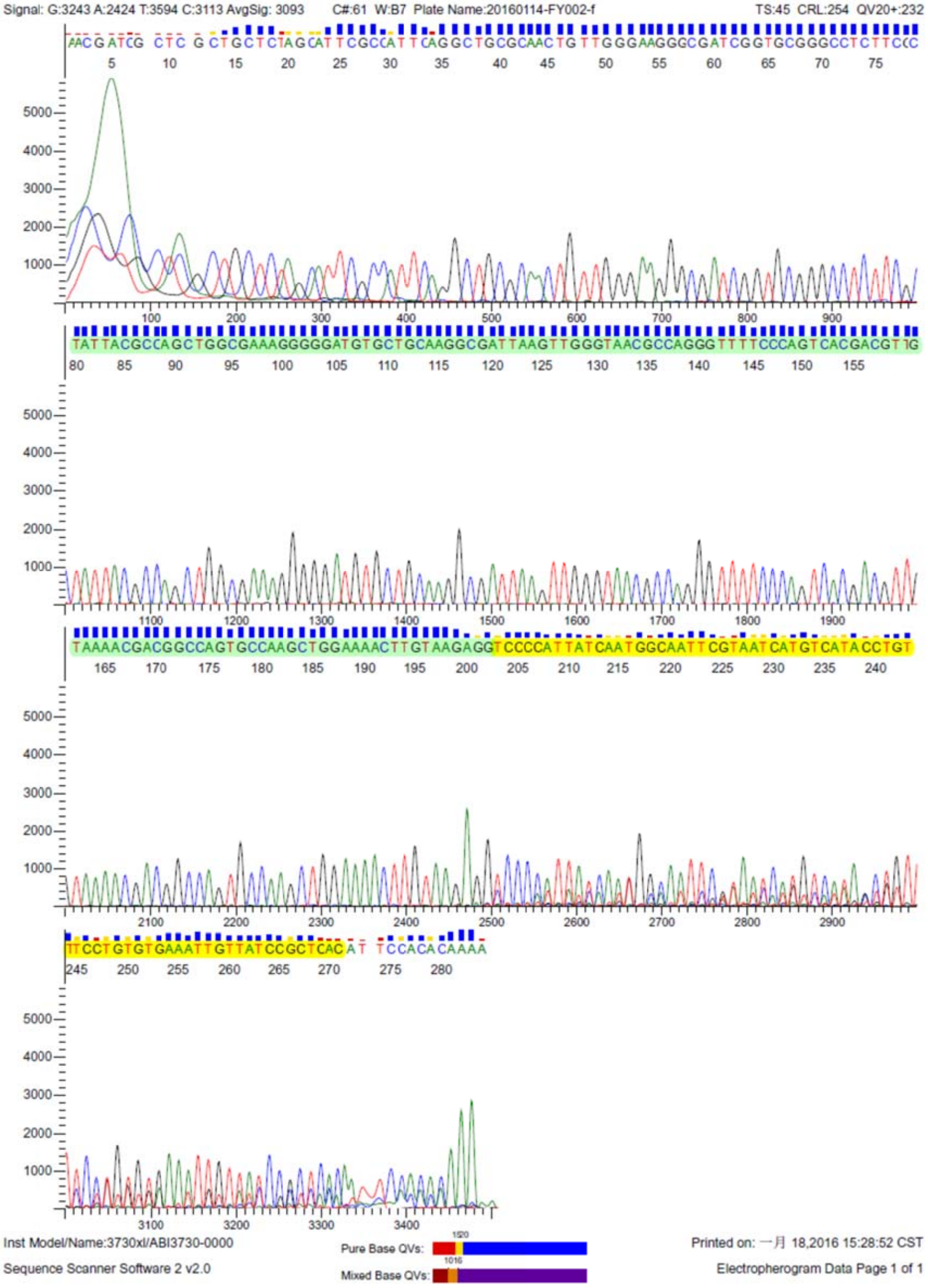

Title : An alternative strategy for targeted gene replacement in plants using a dual-sgRNA/Cas9 design

Authors: Zhao Y., Zhang C., Liu W., Gao W., Liu C., Song G., Li W-X, Mao L., Chen B., Xu Y., Li X., Xie C.

Supplementary File 7. One more biological replicates of the sequencing evidence for harboring re-joining junction site after DNA donor had been target deleted in plants.

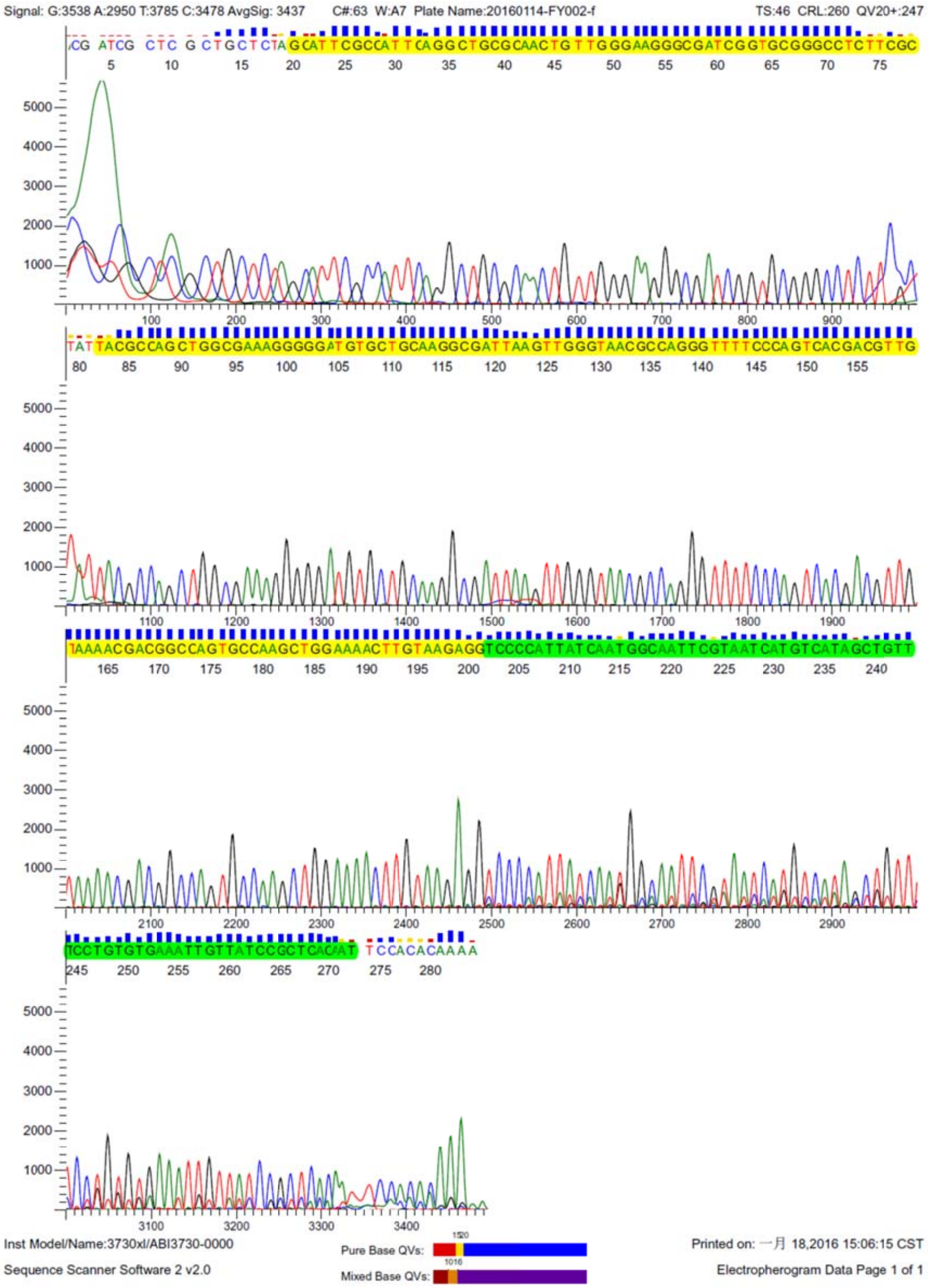

Title : An alternative strategy for targeted gene replacement in plants using a dual-sgRNA/Cas9 design

Authors: Zhao Y., Zhang C., Liu W., Gao W., Liu C., Song G., Li W-X, Mao L., Chen B., Xu Y., Li X., Xie C.

Supplementary File 8. One more biological replicates of the sequencing evidence for harboring re-joining junction site after DNA donor had been target deleted in plants.

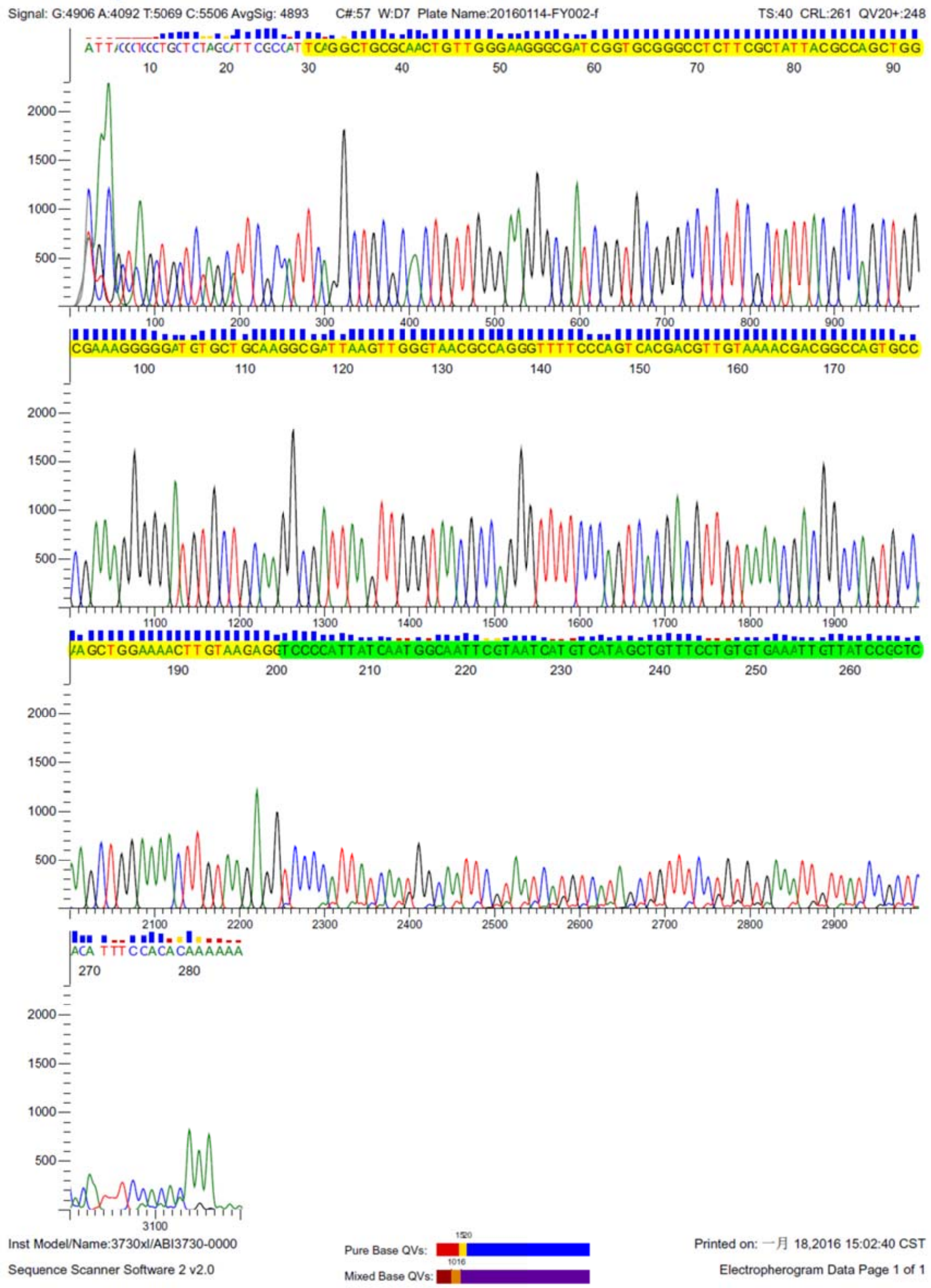

Title : An alternative strategy for targeted gene replacement in plants using a dual-sgRNA/Cas9 design

Authors: Zhao Y., Zhang C., Liu W., Gao W., Liu C., Song G., Li W-X, Mao L., Chen B., Xu Y., Li X., Xie C.

Supplementary File 9. One more biological replicates of the sequencing evidence for harboring re-joining junction site after DNA donor had been target deleted in plants.

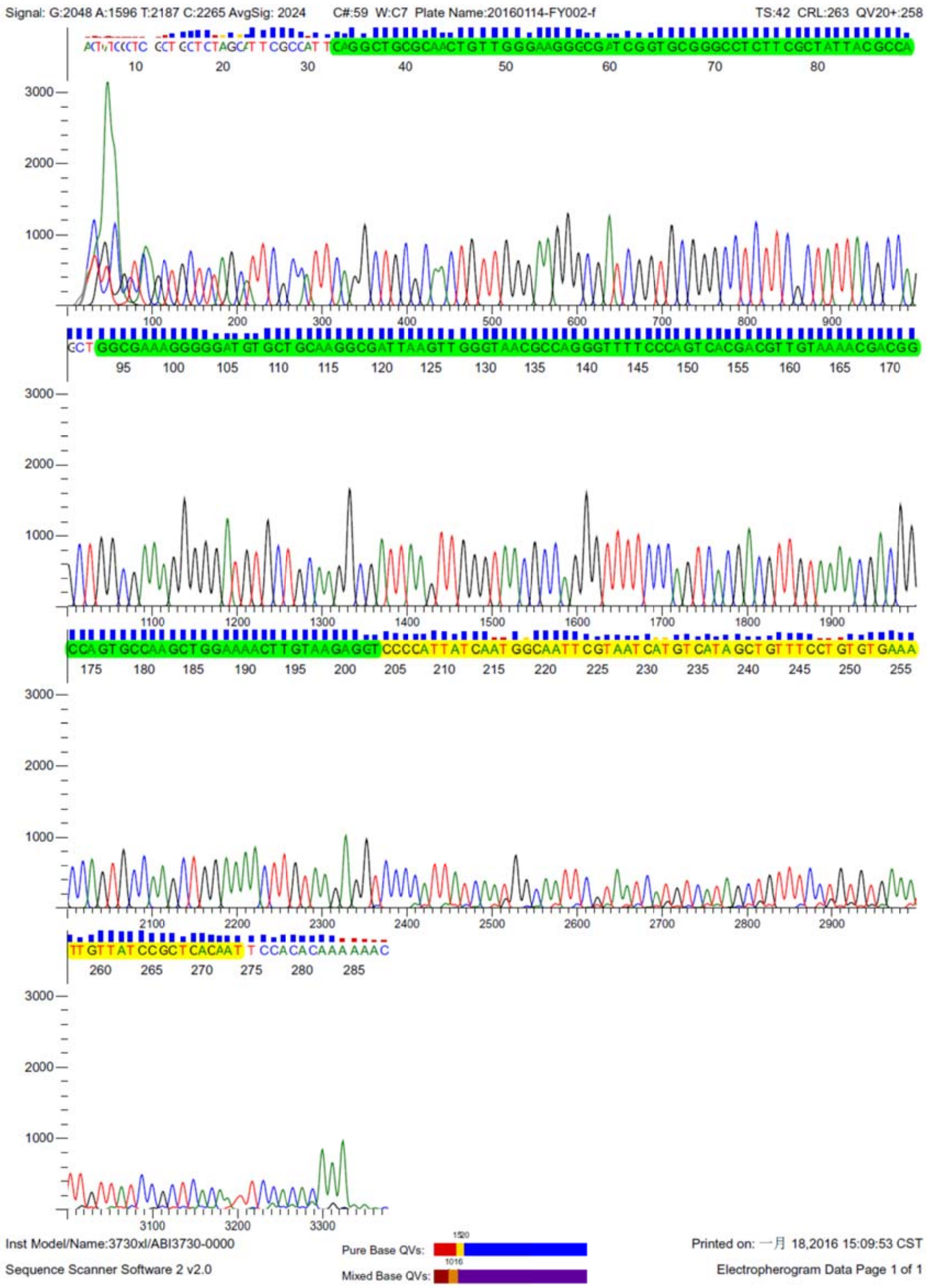

Title ： An alternative strategy for targeted gene replacement in plants using a dual-sgRNA/Cas9 design

Authors： Zhao Y., Zhang C., Liu W., Gao W., Liu C., Song G., Li W-X, Mao L., Chen B., Xu Y., Li X., Xie C.

Supplementary file 10. The sequence of the mentioned key elements of CRISPR/Cas9 expression cassette in this study.

>*hspCas9* sequence

ATGGACTATAAGGACCACGACGGAGACTACAAGGATCATGATATTGATTACAAAGACGATGACGATAAGATGGCCCC
AAAGAAGAAGCGGAAGGTCGGTATCCACGGAGTCCCAGCAGCCGACAAGAAGTACAGCATCGGCCTGGACATCGG
CACCAACTCTGTGGGCTGGGCCGTGATCACCGACGAGTACAAGGTGCCCAGCAAGAAATTCAAGGTGCTGGGCAA
CACCGACCGGCACAGCATCAAGAAGAACCTGATCGGAGCCCTGCTGTTCGACAGCGGCGAAACAGCCGAGGCCAC
CCGGCTGAAGAGAACCGCCAGAAGAAGATACACCAGACGGAAGAACCGGATCTGCTATCTGCAAGAGATCTTCAG
CAACGAGATGGCCAAGGTGGACGACAGCTTCTTCCACAGACTGGAAGAGTCCTTCCTGGTGGAAGAGGATAAGAA
GCACGAGCGGCACCCCATCTTCGGCAACATCGTGGACGAGGTGGCCTACCACGAGAAGTACCCCACCATCTACCAC
CTGAGAAAGAAACTGGTGGACAGCACCGACAAGGCCGACCTGCGGCTGATCTATCTGGCCCTGGCCCACATGATCA
AGTTCCGGGGCCACTTCCTGATCGAGGGCGACCTGAACCCCGACAACAGCGACGTGGACAAGCTGTTCATCCAGCT
GGTGCAGACCTACAACCAGCTGTTCGAGGAAAACCCCATCAACGCCAGCGGCGTGGACGCCAAGGCCATCCTGTC
TGCCAGACTGAGCAAGAGCAGACGGCTGGAAAATCTGATCGCCCAGCTGCCCGGCGAGAAGAAGAATGGCCTGTT
CGGAAACCTGATTGCCCTGAGCCTGGGCCTGACCCCCAACTTCAAGAGCAACTTCGACCTGGCCGAGGATGCCAAA
CTGCAGCTGAGCAAGGACACCTACGACGACGACCTGGACAACCTGCTGGCCCAGATCGGCGACCAGTACGCCGAC
CTGTTTCTGGCCGCCAAGAACCTGTCCGACGCCATCCTGCTGAGCGACATCCTGAGAGTGAACACCGAGATCACCA
AGGCCCCCCTGAGCGCCTCTATGATCAAGAGATACGACGAGCACCACCAGGACCTGACCCTGCTGAAAGCTCTCGT
GCGGCAGCAGCTGCCTGAGAAGTACAAAGAGATTTTCTTCGACCAGAGCAAGAACGGCTACGCCGGCTACATTGAC
GGCGGAGCCAGCCAGGAAGAGTTCTACAAGTTCATCAAGCCCATCCTGGAAAAGATGGACGGCACCGAGGAACTG
CTCGTGAAGCTGAACAGAGAGGACCTGCTGCGGAAGCAGCGGACCTTCGACAACGGCAGCATCCCCCACCAGATC
CACCTGGGAGAGCTGCACGCCATTCTGCGGCGGCAGGAAGATTTTTACCCATTCCTGAAGGACAACCGGGAAAAGA
TCGAGAAGATCCTGACCTTCCGCATCCCCTACTACGTGGGCCCTCTGGCCAGGGGAAACAGCAGATTCGCCTGGAT
GACCAGAAAGAGCGAGGAAACCATCACCCCCTGGAACTTCGAGGAAGTGGTGGACAAGGGCGCTTCCGCCCAGA
GCTTCATCGAGCGGATGACCAACTTCGATAAGAACCTGCCCAACGAGAAGGTGCTGCCCAAGCACAGCCTGCTGTA
CGAGTACTTCACCGTGTATAACGAGCTGACCAAAGTGAAATACGTGACCGAGGGAATGAGAAAGCCCGCCTTCCTG
AGCGGCGAGCAGAAAAAGGCCATCGTGGACCTGCTGTTCAAGACCAACCGGAAAGTGACCGTGAAGCAGCTGAA
AGAGGACTACTTCAAGAAAATCGAGTGCTTCGACTCCGTGGAAATCTCCGGCGTGGAAGATCGGTTCAACGCCTCC
CTGGGCACATACCACGATCTGCTGAAAATTATCAAGGACAAGGACTTCCTGGACAATGAGGAAAACGAGGACATTC
TGGAAGATATCGTGCTGACCCTGACACTGTTTGAGGACAGAGAGATGATCGAGGAACGGCTGAAAACCTATGCCCA
CCTGTTCGACGACAAAGTGATGAAGCAGCTGAAGCGGCGGAGATACACCGGCTGGGGCAGGCTGAGCCGGAAGCT
GATCAACGGCATCCGGGACAAGCAGTCCGGCAAGACAATCCTGGATTTCCTGAAGTCCGACGGCTTCGCCAACAGA
AACTTCATGCAGCTGATCCACGACGACAGCCTGACCTTTAAAGAGGACATCCAGAAAGCCCAGGTGTCCGGCCAGG
GCGATAGCCTGCACGAGCACATTGCCAATCTGGCCGGCAGCCCCGCCATTAAGAAGGGCATCCTGCAGACAGTGAA
GGTGGTGGACGAGCTCGTGAAAGTGATGGGCCGGCACAAGCCCGAGAACATCGTGATCGAAATGGCCAGAGAGAA
CCAGACCACCCAGAAGGGACAGAAGAACAGCCGCGAGAGAATGAAGCGGATCGAAGAGGGCATCAAAGAGCTGG
GCAGCCAGATCCTGAAAGAACACCCCGTGGAAAACACCCAGCTGCAGAACGAGAAGCTGTACCTGTACTACCTGC
AGAATGGGCGGGATATGTACGTGGACCAGGAACTGGACATCAACCGGCTGTCCGACTACGATGTGGACCATATCGTG
CCTCAGAGCTTTCTGAAGGACGACTCCATCGACAACAAGGTGCTGACCAGAAGCGACAAGAACCGGGGCAAGAGC

GACAACGTGCCCTCCGAAGAGGTCGTGAAGAAGATGAAGAACTACTGGCGGCAGCTGCTGAACGCCAAGCTGATT
ACCCAGAGAAAGTTCGACAATCTGACCAAGGCCGAGAGAGGCGGCCTGAGCGAACTGGATAAGGCCGGCTTCATC
AAGAGACAGCTGGTGGAAACCCGGCAGATCACAAAGCACGTGGCACAGATCCTGGACTCCCGGATGAACACTAAG
TACGACGAGAATGACAAGCTGATCCGGGAAGTGAAAGTGATCACCCTGAAGTCCAAGCTGGTGTCCGATTTCCGGA
AGGATTTCCAGTTTTACAAAGTGCGCGAGATCAACAACTACCACCACGCCCACGACGCCTACCTGAACGCCGTCGT
GGGAACCGCCCTGATCAAAAAGTACCCTAAGCTGGAAAGCGAGTTCGTGTACGGCGACTACAAGGTGTACGACGT
GCGGAAGATGATCGCCAAGAGCGAGCAGGAAATCGGCAAGGCTACCGCCAAGTACTTCTTCTACAGCAACATCATG
AACTTTTTCAAGACCGAGATTACCCTGGCCAACGGCGAGATCCGGAAGCGGCCTCTGATCGAGACAAACGGCGAA
ACCGGGGAGATCGTGTGGGATAAGGGCCGGGATTTTGCCACCGTGCGGAAAGTGCTGAGCATGCCCCAAGTGAATA
TCGTGAAAAAGACCGAGGTGCAGACAGGCGGCTTCAGCAAAGAGTCTATCCTGCCCAAGAGGAACAGCGATAAGC
TGATCGCCAGAAAGAAGGACTGGGACCCTAAGAAGTACGGCGGCTTCGACAGCCCCACCGTGGCCTATTCTGTGCT
GGTGGTGGCCAAAGTGGAAAAGGGCAAGTCCAAGAAACTGAAGAGTGTGAAAGAGCTGCTGGGGATCACCATCAT
GGAAAGAAGCAGCTTCGAGAAGAATCCCATCGACTTTCTGGAAGCCAAGGGCTACAAAGAAGTGAAAAAGGACCT
GATCATCAAGCTGCCTAAGTACTCCCTGTTCGAGCTGGAAAACGGCCGGAAGAGAATGCTGGCCTCTGCCGGCGAA
CTGCAGAAGGGAAACGAACTGGCCCTGCCCTCCAAATATGTGAACTTCCTGTACCTGGCCAGCCACTATGAGAAGC
TGAAGGGCTCCCCCGAGGATAATGAGCAGAAACAGCTGTTTGTGGAACAGCACAAGCACTACCTGGACGAGATCAT
CGAGCAGATCAGCGAGTTCTCCAAGAGAGTGATCCTGGCCGACGCTAATCTGGACAAAGTGCTGTCCGCCTACAAC
AAGCACCGGGATAAGCCCATCAGAGAGCAGGCCGAGAATATCATCCACCTGTTTACCCTGACCAATCTGGGAGCCC
CTGCCGCCTTCAAGTACTTTGACACCACCATCGACCGGAAGAGGTACACCAGCACCAAAGAGGTGCTGGACGCCA
CCCTGATCCACCAGAGCATCACCGGCCTGTACGAGACACGGATCGACCTGTCTCAGCTGGGAGGCGACAAAAGGC
CGGCGGCCACGAAAAAGGCCGGCCAGGCAAAAAAGAAAAAGTAA

>AtU6-26 Sequence

CATTCGGAGTTTTTGTATCTTGTTTCATAGTTTGTCCCAGGATTAGAATGATTAGGCATCGAACCTTCAAGAATTTGAT
TGAATAAAACATCTTCATTCTTAAGATATGAAGATAATCTTCAAAAGGCCCCTGGGAATCTGAAAGAAGAGAAGCAG
GCCCATTTATATGGGAAAGAACAATAGTATTTCTTATATAGGCCCATTTAAGTTGAAAACAATCTTCAAAAGTCCCAC
ATCGCTTAGATAAGAAAACGAAGCTGAGTTTATATACAGCTAGAGTCGAAGTAGTGATT

> Enhanced CaMV35S promoter

CCTGCAGGTCAACATGGTGGAGCACGACACACTTGTCTACTCCAAAAATATCAAAGATACAGTCTCAGAAGACCAA
AGGGCAATTGAGACTTTTCAACAAAGGGTAATATCCGGAAACCTCCTCGGATTCCATTGCCCAGCTATCTGTCACTTT
ATTGTGAAGATAGTGGAAAAGGAAGGTGGCTCCTACAAATGCCATCATTGCGATAAAGGAAAGGCCATCGTTGAAG
ATGCCTCTGCCGACAGTGGTCCCAAAGATGGACCCCCACCCACGAGGAGCATCGTGGAAAAAGAAGACGTTCCAA
CCACGTCTTCAAAGCAAGTGGATTGATGTGATAACATGGTGGAGCACGACACACTTGTCTACTCCAAAAATATCAAA
GATACAGTCTCAGAAGACCAAAGGGCAATTGAGACTTTTCAACAAAGGGTAATATCCGGAAACCTCCTCGGATTCC
ATTGCCCAGCTATCTGTCACTTTATTGTGAAGATAGTGGAAAAGGAAGGTGGCTCCTACAAATGCCATCATTGCGATA
AAGGAAAGGCCATCGTTGAAGATGCCTCTGCCGACAGTGGTCCCAAAGATGGACCCCCACCCACGAGGAGCATCG
TGGAAAAAGAAGACGTTCCAACCACGTCTTCAAAGCAAGTGGATTGATGTGATATCTCCACTGACGTAAGGGATGA
CGCACAATCCCACTATCCTTCGCAAGACCCTTCCTCTATATAAGGAAGTTCATTTCATTTGGAGAGGACCTCGACCTC
AACACAACATATACAAAACAAACGAATCTCAAGCAATCAAGCATTCTACTTCTATTGCAGCAATTTAAATCATTTCTT
TTAAAGCAAAAGCAATTTTCTGAAAATTTTCACCATTTACGAACGATA

> SV40 NLS sequence

ATGGCCCCAAAGAAGAAGCGGAAGGTCGGTATCCACGGAGTCCCAGCAGCC

> Nucleoplasmin NLS sequence

AAAAGGCCGGCGGCCACGAAAAAGGCCGGCCAGGCAAAAAAGAAAAAG

> Universal sgRNA sequence

GTTTTAGAGCTAGAAATAGCAAGTTAAAATAAGGCTAGTCCGTTATCAACTTGAAAAAGTGGCACCGAGTCGGTGCT
TTTTTT

Title : An alternative strategy for targeted gene replacement in plants using a dual-sgRNA/Cas9 design

Authors： Zhao Y., Zhang C., Liu W., Gao W., Liu C., Song G., Li W-X, Mao L., Chen B., Xu Y., Li X., Xie C.

Supplementary File 11. The list of primers used in this study.

Gene or target	Primer orientation	Sequence (5'-3')	Application
AtMIR169a	Forward	AGGATGGAGAAGCATGGAGG	PCR screening of deletion mutation
	Reverse	CTCATGGTTGGCAGCAGTTT	
AtMIR827a	Forward	CCTTTTTTTCTGTAATCACCAGT	PCR screening of deletion mutation
	Reverse	AGCTTCAGAGGTTCCAAATACA	
AtU6-26	AtU6-26-1F	GCAGGCATGCAAGCTCATTCGGAGTTTTTGTATCTTGTT	To get sequence for vector construction
	AtU6-26-2F	GCTTTTTTTTAAGCTCATTCGGAGTTTTTGTATCTTGTT	
	Reverse	AATCACTACTTCGACTCTAGCTGTATAT	
Enhanced CaMV35S promoter	Forward	GGCCAGTGCCAAGCTTGCATGCCTGCAGGTCAAC	To get sequence for vector construction
	Reverse	GATCGGGGAAATTCGAGCTCTATCGTTCGTAAATGGTGAAAATT	
AtMIR169a sgRNA1 and sgRNA2	sgRNA1F	ATATACAGCTAGAGTCGAAGTAGTGATTGAGATTTTATGCCCCCAAGAGTTTTAGAGCTAGAAATAGCAAGTT	In fusion PCR for vector construction
	sgRNA2F	ATATACAGCTAGAGTCGAAGTAGTGATTGAAATAGTTTCTAATTCTGGGTTTTAGAGCTAGAAATAGCAAGTT	
	UsgRNA-R	GGCCAGTGCCAAGCTTAAAAAAAAGCACCGACTCG	
AtMIR827a sgRNA1 and sgRNA2	sgRNA1F	ATATACAGCTAGAGTCGAAGTAGTGATTGGATCATCTATTGAAGGAACGTTTTAGAGCTAGAAATAGCAAGTT	In fusion PCR for vector construction
	sgRNA2F	ATATACAGCTAGAGTCGAAGTAGTGATTGCAAATCGAAAAGCTTCTTAGTTTTAGAGCTAGAAATAGCAAGTT	
	UsgRNA-R	GGCCAGTGCCAAGCTTAAAAAAAAGCACCGACTCG	
U6: gRNA1: sgRNA	UgRNA1F	GCAGGCATGCAAGCTCATTCGGAGTTTTTGTATCTTGTT	Splicing overlap extention PCR for vector construction
	UsgRNA-R	GGCCAGTGCCAAGCTTAAAAAAAAGCACCGACTCG	
U6: gRNA2: sgRNA	UgRNA2F	GCTTTTTTTTAAGCTCATTCGGAGTTTTTGTATCTTGTT	Splicing overlap extention PCR for vector construction
	UsgRNA-R	GGCCAGTGCCAAGCTTAAAAAAAAGCACCGACTCG	
AtTFL1	TFL1-F1	CTTTGGTTTTCATTTGGTTATCG	PCR screening of gene replacement and for TA clone of amplicons for sequencing.
	TFL1-R1	AGAGAAAGAGACGACCGAGACAT	
	TFL1-F2	ATGTCTCGGTCGTCTCTTTCTCT	

	TFL1-R2	ATGGTGAGCAAGGGCGAGGAG	
	TFL1-F3	GCAGAGGCATCTTCAACGAT	
	TFL1-R3	CATGACCTGTTTTGCATTTCG	
	TFL1-F4	CGAAATGCAAAACAGGTCATG	
	TFL1-R4	GTGACCTATCAAGCCATGTATGAG	
	F5 (BEcoRI-F)	TTGTGTGGAATTGTGAGCGG	PCR identification of DNA donor had been deleted and TA clone for sequencing validation.
	R5 (AHindIII-R)	AAACTGAAGGCGGGAAACG	
M13 R		CAGGAAACAGCTATGAC	Sequencing mutations in TA clone

Title：An alternative strategy for targeted gene replacement in plants using a dual-sgRNA/Cas9 design

Authors： Zhao Y., Zhang C., Liu W., Gao W., Liu C., Song G., Li W-X, Mao L., Chen B., Xu Y., Li X., Xie C.

Supplementary File 12. The sequence detail of DNA donor template before and after deletion to supply for HDR repair.

Note:

- The DNA repair donor sequence should be the sequence between 1st and 2nd sgRNA target sites. This sequence would be deleted to supply as DNA repair donor
- The two ↓ indicating the expected cut sites induced by Cas9 nuclease.
- PAM (protospacer-adjacent motif) sequences had been underlined and indicated as "PAM".
- This sequence harboring left region and right border (RB) sequence, eGFP expression cassette and both flanking homolog arms of TFL1.
- The text between the lines of the sequence were the captions.
- Detection primer pair (F5 and R5 pair were designed on the RE site of the *Eco*RI and *Hind*III, respectively.)
  F5 (BEcoRI-F): 5' TTGTGTGGAATTGTGAGCGG 3'
  R5 (AHindIII-R): 5' AAACTGAAGGCGGGAAACG 3'
  the blue shadow indicating the sequence regions.

I. Before deletion happened (total length 3902 bp)

> left region outside repair donor (1-96 bp region)

Primer F5 (BEcoRI-F)

AGGCTTTACA CTTTATGCTT CCGGCTCGTA TGTTGTGTGG AATTGTGAGC GGATAACAATTTCACACAGG
AAACAGCTAT GACATGATTA CGAATT

>TFL1 left homologous arm (97-897 bp region)

1st sgRNA target region PAM

GCCATTGATA ATGGGGA ↓ GAG TGGTTAGCGG TTTTTTTGCC CAGGCCTACT CTGAGCAATA
ATTGTATCCG GAGTTGTAAT AGAATCAAAG TACGATGAGA GTGTTTTTAT GACAAATATC
TTAATCTTGG CCAATTATAT GTTCTACTGA AATTCTTTTT GAATTCATCG ACCAGTGAGA
CTTAAAAATA GCTTTTTATT CGCCGAGGTA TATATAGCTA GGAATTTTGT CGAAATTTAG
ACGTTAGTGG GTTTTGTTCT TCGTGACACA AAAGATATTC TATATATTAA CGAAATCTAG
CGATCGATAT GGTATTTATA TAAAGTCTTG GTCATAGATA GGGGTTGAAA CTTGAAACCA
TGCATGATAT GCCAATGTTG CTGAAGCAGT CAATGTTGCT GAAGAAGTCA AACGTAATTA
TATAGTGAAT ACCAAAAAAG TGATATTTCT TAATTCAATT AAATATAATT ATAGTTTTAA
ATCACCTAAA ATAAGTTACT TATTAAAACC CCCCAAATTT ACTTTAATAT AGTTGGTGTA
CATGTTTGAG AAAGCAAACA AAAAGAAAAA GAAAAAGAAA AAAAAAAGAG AAAGAGGTTA
GTACACATAA TTGGGAATTA ATGTCTATTG ATTCTTTTAT CTTTCTCTCT CTCTCTAAGA
CGGAAAACCC CTATAAATAG ATGTCTCGGT CGTCTCTTTG TCTCCCAAAT CACTACAAAT
CTCTCTTTTC CTCTAAGTTA ACAAAAGAAA ATGGAGAATA TGGGAACTAG AGTGATAGAG
CCATTGATAA TGGGGAAATT C

〉Tnos-eGFP-E35Spromoter (898-2812 bp region, the expression cassettee was placed on the minus strand）

CCGATCTAGT AACATAGATG ACACCGCGCG CGATAATTTA TCCTAGTTTG
CGCGCTATAT TTTGTTTTCT ATCGCGTATT AAATGTATAA TTGCGGGACT
CTAATCATAA AAACCCATCT CATAAATAAC GTCATGCATT ACATGTTAAT
TATTACATGC TTAACGTAAT TCAACAGAAA TTATATGATA ATCATCGCAA
GACCGGCAAC AGGATTCAAT CTTAAGAAAC TTTATTGCCA AATGTTTGAA
CGATCGGGGA AATTCGAGCT CGGTACCCGG GCGATCATAC CTTTCTCTTC
TTCTTGGGAG AACCCCCTTT GTACAGCTCG TCCATGCCGT GAGTGATCCC
GGCGGCGGTC ACGAACTCCA GCAGGACCAT GTGATCGCGC TTCTCGTTGG
GGTCTTTGCT CAGGGCGGAC TGGGTGCTCA GGTAGTGGTT GTCGGGCAGC
AGCACGGGGC CGTCGCCGAT GGGGGTGTTC TGCTGGTAGT GGTCGGCGAG
CTGCACGCTG CCGTCCTCGA TGTTGTGGCG GATCTTGAAG TTCACCTTGA
TGCCGTTCTT CTGCTTGTCG GCCATGATAT AGACGTTGTG GCTGTTGTAG
TTGTACTCCA GCTTGTGCCC CAGGATGTTG CCGTCCTCCT TGAAGTCGAT
GCCCTTCAGC TCGATGCGGT TCACCAGGGT GTCGCCCTCG AACTTCACCT
CGGCGCGGGT CTTGTAGTTG CCGTCGTCCT TGAAGAAGAT GGTGCGCTCC
TGGACGTAGC CTTCGGGCAT GGCGGACTTG AAGAAGTCGT GCTGCTTCAT
GTGGTCGGGG TAGCGGCTGA AGCACTGCAC GCCGTAGGTG AAGGTGGTCA
CGAGGGTGGG CCAGGGCACG GGCAGCTTGC CGGTGGTGCA GATGAACTTC
AGGGTCAGCT TGCCGTAGGT GGCATCGCCC TCGCCCTCGC CGGACACGCT
GAACTTGTGG CCGTTTACGT CGCCGTCCAG CTCGACCAGG ATGGGCACCA
CCCCGGTGAA CAGCTCCTCG CCCTTGCTCA CCATCCCGGG GATCCTCTAG
AGTCCCCCGT GTTCTCTCCA AATGAAATGA ACTTCCTTAT ATAGAGGAAG
GGTCTTGCGA AGGATAGTGG GATTGTGCGT CATCCCTTAC GTCAGTGGAG
ATATCACATC AATCCACTTG CTTTGAAGAC GTGGTTGGAA CGTCTTCTTT
TTCCACGATG CTCCTCGTGG GTGGGGGTCC ATCTTTGGGA CCACTGTCGG

CAGAGGCATC TTCAACGATG GCCTTTCCTT TATCGCAATG ATGGCATTTG
TAGGAGCCAC CTTCCTTTTC CACTATCTTC ACAATAAAGT GACAGATAGC
TGGGCAATGG AATCCGAGGA GGTTTCCGGA TATTACCCTT TGTTGAAAAG
TCTCAATTGC CCTTTGGTCT TCTGAGACTG TATCTTTGAT ATTTTTGGAG
TAGACAAGTG TGTCGTGCTC CACCATGTTG ACGAAGATTT TCTTCTTGTC
ATTGAGTCGT AAGAGACTCT GTATGAACTG TTCGCCAGTC TTTACGGCGA
GTTCTGTTAG GTCCTCTATT TGAATCTTTG ACTCCATGGC CTTTGATTCA
GTGGGAACTA CCTTTTTAGA GACTCCAATC TCTATTACTT GCCTTGGTTT
GTGAAGCAAG CCTTGAATCG TCCATACTGG AATAGTACTT CTGATCTTGA
GAAATATATC TTTCTCTGTG TTCTTGATGC AGTTAGTCCT GAATCTTTTG
ACTGCATCTT TAACCTTCTT GGGAAGGTAT TTGATTTCCT GGAGATTATT
GCTCGGGTAG ATCGTCTTGA TGAGACCTGC TGCGTAAGCC TCTCTAACCA
TCTGTGGGTT AGCATTCTTT CTGAAATTGA AAAGGCTAAT CTGGGGACCT
GCAGGCATGC AAGCT

> TFL1 right homologous arm (2813-3665 bp region)

CCTCTTACAA GTTTTCCATT TCTAACTCAA TAATCTTATA AATTGTAGCT TTAGTTTTTA
TCATTCCTTT TTCCAGTCTT TTTTTTTTAA TGGTAAAACT CAACCGAAAT GCAAAACAGG
TGATGATAGA CCCAGATGTT CCAGGTCCTA GTGACCCCTT TCTAAAAGAA CACCTGCACT
GGTACGTTTA ATTTATTTAT TCTTTCTTTT CATTTTGGGC CCATATTCCA TATACATTGC
ATTTAAATCA TTTCGTTATA ACCCTAATAA AGTTTTTTTT GGGTGTAAGT TATATACATT
TGAGTTGGTC AAAGATCTCC ATCGCCATGA GTTCTCAGAA CTTTTTCTGT AAAGTAATAA
TATTAGTATT GTTGAATGTT TCAATAGGAT CGTTACAAAC ATTCCCGGCA CAACAGATGC
TACGTTTGGT AAGGCCTCTT CATGAATCTT GTAATTTAAA TACTTATACA TATATCATGT
TATATAGAAA TAAAAATATT TGCATTGTAA TATAGGCAAA GAGGTGGTGA GCTATGAATT
GCCAAGGCCA AGCATAGGGA TACATAGGTT TGTGTTTGTT CTGTTCAGGC AGAAGCAAAG
ACGTGTTATC TTTCCTAATA TCCCTTCGAG AGATCACTTC AACACTCGTA AATTTGCGGT
CGAGTATGAT CTTGGTCTCC CTGTCGCGGC CGTCTTCTTT AACGCACAAA GAGAAACCGC
TGCACGCAAA CGCTAGTTTC ATGATTGTCA TAAACTGCAA AAATGAAAGA AGAAAATTTG
CATGTAATCT CATGTTTATT TGTGTTCTGA ATTTCCGTAC TCTGACCTTT T ↓ CCTCTTACA
PAM 2nd sgRNA target region
AGTTTTCCAG CTT

> right border (3667-3902 bp region)

GGCACTG GCCGTCGTTT TACAACGTCG TGACTGGGAA AACCCTGGCG TTACCCAACT TAATCGCCTT
GCAGCACATC CCCCTTTCGC CAGCTGGCGT AATAGCGAAG AGGCCCGCAC CGATCGCCCT TCCCAACAGT
TGCGCAGCCT GAATGGCGAA TGCTAGAGCA GCTTGAGCTT GGATCAGATT GTCGTTTCCC GCCTTCAGTT
TAAACTATCA GTGTTGACAG TAATTGGGCG R5 (AHindIII-R)

II. After deletion happened to supply the DNA donor for HDR repair

1> The junction sequence after donor template has been cut to supply for HDR repair

TTGTGTGGAA TTGTGAGCGG ATAACAATTT CACACAGGAA ACAGCTATGA CATGATTACG
AATTGCCATT GATAATGGGG ACCTCTTACA AGTTTTCCAG CTTGGCACTG GCCGTCGTTT
The junction site
TACAACGTCG TGACTGGGAA AACCCTGGCG TTACCCAACT TAATCGCCTT GCAGCACATC
CCCCTTTCGC CAGCTGGCGT AATAGCGAAG AGGCCCGCAC CGATCGCCCT TCCCAACAGT
TGCGCAGCCT GAATGGCGAA TGCTAGAGCA GCTTGAGCTT GGATCAGATT GTCGTTTCCC
GCCTTCAGTT T

2> Reverse complementary of the Junction sequence

AAACTGAAGG CGGGAAACGA CAATCTGATC CAAGCTCAAG CTGCTCTAGC
ATTCGCCATT CAGGCTGCGC AACTGTTGGG AAGGGCGATC GGTGCGGGCC
TCTTCGCTAT TACGCCAGCT GGCGAAAGGG GGATGTGCTG CAAGGCGATT
AAGTTGGGTA ACGCCAGGGT TTTCCCAGTC ACGACGTTGT AAAACGACGG
CCAGTGCCAA GCTGGAAAAC TTGTAAGAGG TCCCCATTAT CAATGGCAAT
The junction site
TCGTAATCAT GTCATAGCTG TTTCCTGTGT GAAATTGTTA TCCGCTCACA
ATTCCACACA A

Note: Our sequencing results confirmed by sequencing of reverse complementary sequence by using M13 R primer in TA clone of PCR amplicons.
